# NMDA receptors mediate synaptic depression, but not spine loss in the dentate gyrus of adult amyloid Beta (Aβ) overexpressing mice

**DOI:** 10.1186/s40478-018-0611-4

**Published:** 2018-10-23

**Authors:** Michaela Kerstin Müller, Eric Jacobi, Kenji Sakimura, Roberto Malinow, Jakob von Engelhardt

**Affiliations:** 1 Institute of Pathophysiology, University Medical Center of the Johannes Gutenberg University Mainz, 55128 Mainz, Germany; 2 Synaptic Signalling and Neurodegeneration, German Center for Neurodegenerative Diseases (DZNE), 53127 Bonn, Germany; 3 Department of Cellular Neurobiology, Brain Research Institute, Niigata University, Niigata, 951-8585 Japan; 4 Center for Neural Circuits and Behavior, Department of Neuroscience and Section for Neurobiology, Division of Biology, University of California at San Diego, San Diego, CA USA

**Keywords:** NMDA receptor, Amyloid Beta, Alzheimer’s disease, GluN2B, GluN2A

## Abstract

Amyloid beta (Aβ)-mediated synapse dysfunction and spine loss are considered to be early events in Alzheimer’s disease (AD) pathogenesis. *N*-methyl-D-aspartate receptors (NMDARs) have previously been suggested to play a role for Amyloid beta (Aβ) toxicity. Pharmacological block of NMDAR subunits in cultured neurons and mice suggested that NMDARs containing the GluN2B subunit are necessary for Aβ-mediated changes in synapse number and function in hippocampal neurons. Interestingly, NMDARs undergo a developmental switch from GluN2B- to GluN2A-containing receptors. This indicates different functional roles of NMDARs in young mice compared to older animals. In addition, the lack of pharmacological tools to efficiently dissect the role of NMDARs containing the different subunits complicates the interpretation of their specific role. In order to address this problem and to investigate the specific role for Aβ toxicity of the distinct NMDAR subunits in dentate gyrus granule cells of adult mice, we used conditional knockout mouse lines for the subunits GluN1, GluN2A and GluN2B. Aβ-mediated changes in synaptic function and neuronal anatomy were investigated in several-months old mice with virus-mediated overproduction of Aβ and in 1-year old 5xFAD mice. We found that all three NMDAR subunits contribute to the Aβ-mediated decrease in the number of functional synapses. However, NMDARs are not required for the spine number reduction in dentate gyrus granule cells after chronic Aβ-overproduction in 5xFAD mice. Furthermore, the amplitude of synaptic and extrasynaptic NMDAR-mediated currents was reduced in dentate gyrus granule of 5xFAD mice without changes in current kinetics, suggesting that a redistribution or change in subunit composition of NMDARs does not play a role in mediating Amyloid beta (Aβ) toxicity. Our study indicates that NMDARs are involved in AD pathogenesis by compromising synapse function but not by affecting neuron morphology.

**Electronic supplementary material:**

The online version of this article (10.1186/s40478-018-0611-4) contains supplementary material, which is available to authorized users.

## Introduction

Amyloid beta (Aβ) deposition in the brain of Alzheimer Disease (AD) patients initiates a cascade of events that trigger synaptic dysfunction, spine loss and ultimately neuronal death (reviewed in [26]). Indeed, the amount of soluble Aβ correlates highly with the state of cognitive impairment in AD patients [49, 52, 58, 97]. However, despite intense research, it is not well understood how Aβ induces early disease pathologies.

Several studies suggested that Aβ-toxicity is mediated via an influence on NMDAR function or expression [29, 36, 37, 79]. NMDARs are known to play an important role for synaptic plasticity in the healthy brain (reviewed in [88]). Therefore, it has been speculated that altered NMDAR signalling is involved in the pathogenesis of several neurological diseases including AD (reviewed in [42]). Consistently, one of the two types of FDA (U.S. Food and Drug Administration) approved AD therapies targets NMDARs. Thus, the partial NMDAR antagonist Memantine alleviates cognitive impairments in moderate-severe AD patients [68, 73, 83, 100]. However, antagonists that are selective for specific NMDAR subunits would be more effective as AD treatment than the unselective blocker Memantine.

NMDARs are tetramers composed of two obligatory GluN1 subunits and combinations of subunits GluN2A-D and/or GluN3A-B subunits [12, 39, 56]. NMDARs containing different GluN2 subunits differ in their expression profile and function [57, 91, 97]. GluN1, GluN2A and GluN2B are the predominant subunits that are expressed in excitatory neurons of the adult rodent forebrain [57, 98], forming diheteromeric GluN1/GluN2A- and GluN1/GluN2B- as well as triheteromeric GluN1/GluN2A/GluN2B containing NMDARs [50, 77, 86]. The GluN2A subunit is postnatally upregulated [57] and thought to be the major synaptic subunit of homomeric NMDARs of excitatory forebrain neurons in adult mice. In contrast, the GluN2B subunit is also expressed in forebrain neurons of newborn mice, but thought to be present in the majority of extrasynaptic NMDARs [18, 24, 27, 63, 87]. The activation of synaptic NMDARs has been shown to exert protective function [25]. In contrast, activation of extrasynaptic NMDARs activates apoptotic signalling cascades [25, 78].

It has been shown that the GluN2B subunit is involved in the Aβ-mediated synaptic dysfunction and spine loss of cultured neurons [7, 30, 40, 74, 79]. However, studies on Aβ-toxicity in cultured neurons that are prepared from newborn mice may well overestimate the contribution of the GluN2B subunit since they predominantly express this subunit [55, 91, 92]. However, blockade of NMDARs with ifenprodil or radiprodil, antagonists specific for diheteromeric GluN1/GluN2B-containing NMDARs, or deletion of the GluN2B subunit rescued Aβ-induced long-term-potentiation (LTP) deficits [31, 64–66, 70]. This suggests that the GluN2B subunit plays a role for Aβ-toxicity also in the adult brain. It remains to be shown if the GluN2B subunit is also involved in other alterations that are known to be mediated by Aβ-overproduction like changes in basal synaptic function and in the morphology of neurons such as in spine loss, since contrasting data have been published [30, 41, 66, 79, 82].

Little is known about the mechanisms how NMDARs are involved in Aβ-toxicity. Several mechanisms have been proposed including that Aβ may directly bind to NMDARs and influence their gating [14, 43]. Additionally, Aβ-mediated Calcium-influx via NMDARs leads to the formation of reactive oxygen species (ROS) and initializes oxidative stress [13]. An alternative hypothesis suggests that an Aβ-mediated redistribution of NMDARs may increase the vulnerability of neurons to higher extracellular glutamate levels, similar to what has been shown for Huntington’s disease [53]. Thus, a relative upregulation of the number of extrasynaptic GluN2B-containig NMDARs, which activate apoptotic pathways [47, 71, 87], and downregulation of, eventually more neuroprotective, synaptic GluN2A-containing NMDARs [8, 47, 89], could explain an increased susceptibility to excitotoxicity. Aβ indeed decreases NMDAR expression on the cell surface of neurons from post-mortem AD patients [33, 35, 54, 72]. However, it is not clear which NMDAR subunit is affected, whether synaptic or extrasynaptic NMDARs are downregulated, and finally if Aβ induces changes in NMDAR distribution in the adult brain.

To investigate the role of NMDARs for Aβ-toxicity in adult mice, we used conditional NMDAR knockout mice. Changes in synaptic function and neuronal morphology in response to subacute Aβ-overproduction was investigated by a virus-mediated expression of Aβ for several weeks in dentate gyrus (DG) granule cells of adult mice. The DG was chosen as region of interest, since LTP, which is inhibited by Aβ, occurs in CA1 and DG. Since Aβ plaques form in the DG before they appear in CA1 area, we focused on this brain area. The influence of chronic Aβ overproduction was investigated in 1-year old 5xFAD mice. We found that NMDARs indeed play a major role for the influence of Aβ on the number of functional synapses, but not on the Aβ-mediated change in spine number after chronic Aβ overproduction. Moreover, Aβ reduces the expression of NMDARs at both synaptic and extrasynaptic sites without a major influence on subunit composition.

## Material & Methods

### Animals

Mouse experiments were performed according to the German Animal Welfare Act and the Regierungspräsidium Karlsruhe as well as the Landesuntersuchungsamt Rhineland-Palatinate. All procedures followed the “Principles of laboratory animal care” (NIH publication No. 86–23, revised 1985). Mice had access to food and water ad libitum. The conditional NMDAR knockout mouse lines GluN1^fl/fl^ [59], GluN2A ^fl/fl^ [19] and GluN2B^fl/fl^ [60, 93], in which the *grin1, grin2a* and *grin2b* genes are flanked by loxP sites, were used for conditional deletion of the different NMDAR subunits. Mice of both sexes were used. The 5xFAD mouse line [60] was used as a mouse model for AD and crossbred with the conditional NMDAR knockout mice lines. Only female mice were used from these mouse lines. Deletion of NMDAR subunits in the conditional knockout mice was achieved by injection of recombinant adeno-associated viruses (rAAVs) expressing Cre-recombinase into the DG (rAAV-Syn-Cre-T2A-EGFP).

### rAAV production and stereotactic injection

pAAV-CaMKII-T2A-tdTom plasmid was used to subclone Aβ overexpressing DNA (C-terminal 100 (CT100)) from a sindbis virus backbone [36] in an rAAV vector.

**Fig. 1:**
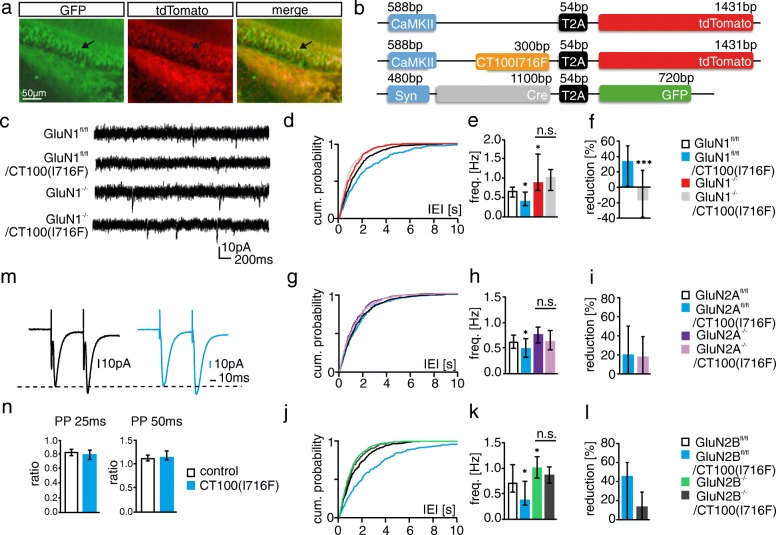
CT100(I716F)-mediated synaptic depression in granule cells of adult mice is NMDAR dependent. **a** Double infection with rAAV-Syn-Cre-T2A-GFP and rAAV-CaMKII-CT100(I716F)-T2A-tdTomato in DG neurons. The arrowhead points to a double-infected DG granule cell. **b** pAAV constructs were used to express CT100(I716F) or Cre-recombinase or tdTomato as control. **c** Example traces of mEPSC recordings from GluN1^fl/fl^ mice injected with the different AAV constructs as indicated. **d + e** CT100(I716F) increases inter-event-interval (IEI) and reduces mEPSC frequency in DG granule cells in cells of GluN1^fl/fl^ mice. Deletion of GluN1 (GluN1^−/−^) increases mEPSC frequency. Overexpression of CT100(I716F) does not significantly reduce mEPSC frequency in GluN1^−/−^ granule cells. **f** To test if the effect of CT100(I716F) in GluN1^fl/fl^ neurons is different from that in GluN1^−/−^ granule cells, we calculated the respective percent of CT100(I716F)-mediated reduction in mEPSC frequency. The mEPSC frequency is smaller in GluN1^fl/fl^/CT100(I716F) than in GluN1^fl/fl^ cells (blue bar) and slightly bigger in GluN1^−/−^/CT100(I716F) than in GluN1^−/−^ cells (gray bar). The reduction in GluN1^fl/fl^ cells is significantly bigger than the effect of CT100(I716F) in GluN1^−/−^ granule cells. **g + h** CT100(I716F) reduces mEPSC frequency in DG granule cells in cells of GluN2A^fl/fl^ mice, but does not significantly reduce mEPSC frequency in GluN2A^−/−^ granule cells. **i** The CT100(I716F)-mediated decrease in mEPSC frequency in GluN2A^fl/fl^ cells is not significantly different from the decrease in GluN2A^−/−^ cells. **j + k** CT100(I716F) increases IEI and reduces mEPSC frequency in DG granule cells of GluN2B^fl/fl^ mice. Deletion of GluN2B (GluN2B^−/−^) increases mEPSC frequency. Overexpression of CT100(I716F) does not significantly reduce mEPSC frequency in GluN2B^−/−^ granule cells. **l** The CT100(I716F)-mediated decrease in mEPSC frequency in GluN2B^fl/fl^ cells is not significantly different from the decrease in GluN2B^−/−^ cells. **m** Example traces of paired-pulse recordings (PPR) with pairs of inter-stimulus intervals (ISI) of 25 ms **n** The PPR of the amplitudes of two currents evoked with 25 ms or 50 ms ISIs is not different in control cells and CT100(I716F)-overexpressing cells. ISIs are shown on the top of the quantification. Bar graphs show median ± IQR. * = *p* < 0.05, ** = *p* < 0.01, *** = *p* < 0.001; cum. = cumulative

A mutated CT100 DNA construct containing an isoleucine to phenylalanine switch at amino acid position 716, named CT100(I716F), was constructed via site-directed-mutagenesis (Quik Change II kit from Agilent Technologies, USA) from the pAAV-CaMKII-CT100-T2A-tdTom plasmid to produce pAAV-CaMKII-CT100(I716F)-T2A-tdTom for increased Aβ_42/40_ overexpression [21]. The following constructs were expressed in rAAVs and used in the study: rAAV-CaMKII-tdTom (control cells), rAAV-CaMKII-CT100/CT100(I716F)-T2A-tdTom (CT100 or CT100(I716F) overexpression), rAAV-Syn-Cre-T2A-GFP (NMDAR subunit deletion) and rAAV-Syn-Cre-T2A-GFP + rAAV-CaMKII-CT100/CT100(I716F)-T2A-tdTom (NMDAR subunit deletion and CT100 or CT100(I716F) overexpression) (Fig. 1b and Additional file 1: S1b). Co-injection of control- and Cre-expressing-rAAVs could thus be differentiated by red and green fluorescence (Fig. 1a).

Plasmids used for rAAV1/2 production were amplified with the Qiagen Maxi Kit Plus (Qiagen, Germany). HEK293T cells were transfected with the DNA plasmids with a standard CaCl_2_ transfection protocol and the rAAV was purified via heparin columns (GE Healthcare, England) using standard procedures.

rAAVs were stereotactically injected into the DG through a thin glass capillary using the following coordinates according to bregma: anteroposterior, − 3 mm; mediolateral, ±3 mm; dorsoventral, − 3.5 mm from the skull surface.

#### Preparation of acute slices

Mice were deeply anesthetized with 3% isoflurane and cardially perfused with ice-cold slicing solution (212 mM sucrose, 26 mM NaHCO_3_, 1.25 mM NaH_2_PO_4_, 3 mM KCl, 0.2 mM CaCl_2_, 7 mM MgCl_2_ and 10 mM glucose). Brains were quickly removed and 250 μm thick acute transverse slices were cut in ice-cold slicing Solution with the help of a tissue slicer (slicer: Sigmann Elektronik, Germany; razor blade: Personna, USA). Acute brain slices were immediately transferred to a slice holding chamber with 37 °C ACSF (125 mM NaCl, 25 mM NaHCO_3_, 1.25 mM NaH_2_PO_4_, 2.5 mM KCl, 2 mM CaCl_2_,1 mM MgCl_2_ and 25 mM glucose) and incubated for 15 min. The holding chamber was slowly cooled down to RT and slices were incubated for 45 min before being used in experiments.

### Electrophysiology

Acute transverse slices were completely submerged and continuously perfused with carbogen-saturated artificial cerebral spine fluid (ACSF, see supplemental methods) at RT with a flow-rate of 1 ml/min. Slices were imaged with an Olympus BX51WI upright microscope (Olympus, Japan) fitted with a 4× air (Plan N, NA 0.1; Olympus, Japan) and 40× water-immersion (LUMPlan FI/IR, NA 0.8w; Olympus, Japan) objective. Electrical signals were acquired at 10 kHz for miniature excitatory post-synaptic current (mEPSC) recordings and 50 kHz for all other recordings using an EPC10 amplifier (HEKA, Germany), connected to a probe and PC. Electrical signals were recorded with the help of Patchmaster software (HEKA, Germany). No correction for liquid junction potential was done. For A/N ratios, paired pulse ratio recordings and firing patterns, 10 μM SR95531 hydrobromide (Biotrend, Germany) were added to the ACSF. 1 μM TTX (Biotrend, Germany) and 50 μM APV (Biotrend, Germany) were additionally added in mEPSC recordings. For NMDAR decay experiments 10 μM SR95531 hydrobromide was added with 50 μM CNQX.

For extracellular stimulation of the medial perforant path, the stimulus was generated by a stimulus isolator (WPI, USA) connected with the EPC10 amplifier and triggered by the Patchmaster software. A chlorinated silver wire located inside a borosilicate glass capillary filled with ACSF was used as stimulation electrode. For nucleated patches, cells were slowly pulled out of the slice while simultaneously applying negative pressure after reaching the whole cell configuration. Thus, the nucleus covered with cell membrane was pulled out of the slice and navigated in front of a theta glass tubing mounted onto a piezo translator (PI, Germany). A 1 ms pulse of 1 mM glutamate application solution (in mM): 135 NaCl, 10 HEPES, 5.4 KCl, 1.8 CaCl2, 5 glucose, 0.01 CNQX, 0.01 glycine (pH 7.2) was applied via one pipe of the theta glass. The other pipe contained the application solution without glutamate.

### Morphological analysis

Cells used for morphological analysis were filled with an intracellular solution containing 0.1–0.5% biocytin (Sigma Aldrich, USA) through the patch-pipette while recording. Acute slices were fixed in 4% Histofix (Carl Roth, Germany) after recording. 2–10 days later, slices were washed in 1× PBS (phosphate buffered saline), permeabilized in 0.2% PBST (0.2% Triton in 1× PBS) and stained overnight with a Streptavidin-coupled Alexa594-conjugated antibody (life technologies, USA). Slices from 5xFAD mice were additionally stained with an Alexa488-coupled 6E10 Antibody (Covance, USA) for Aβ plaque staining. After washing in 1× PBS, slices were mounted in ProLong Gold Antifade (life technologies, USA).

Neurons were imaged with a fixed-stage Leica TCS SP5 II microscope (Leica, Germany) and the Leica LAS AF Lite Software (Leica, Germany). Z-stacks from whole neurons were imaged with a 40× oil-immersion objective (Leica, Germany) with the following parameters: voxel size x/y = 0.758 μm, z = 0.209 μm. Z-stacks from dendrites were taken with a 63× oil-immersion objective (Leica, Germany) with the following parameters: voxel size x/y = 0.08 μm, z = 0.168 μm.

Z-stacks of DG granule cells were semi-automatically traced with Neuronstudio (CNIC, Mount Sinai School of Medicine, USA) and Sholl analysis was performed. Dendritic spines were also counted semi-automatically with Neuronstudio.

#### Analysis and statistics

Analysis of electrophysiological experiments was carried out using Clampfit (Molecular Devices, USA), IGOR Pro (WaveMetrix, USA), Microsoft Office Excel (Microsoft, USA) and GraphPad Prism (GraphPad software, USA). For mEPSC analysis, the minianalysis plugin of the Clampfit software was used. For Firing pattern and NMDAR decay analysis, IGOR Pro was used with the Patcher’s Power Tools and Neuromatic analysis package (MPI for biophysical chemistry, Germany and Jason Rothman, http://www.neuromatic.thinkrandom.com/). Morphological datasets were analyzed using the NeuronStudio software. Amira (FEI, USA) was used for blind deconvolution to improve image quality for spine analysis.

Statistics were performed with Graphpad Prism 6 (Graphpad, USA). Sholl analysis was statistically evaluated with a two-way ANOVA (analysis of variance with Tukey test for multiple comparisons). Datasets were tested for statistical significance with Mann-Whitney (MW) or Kruskal-Wallis (followed by Dunn’s posttest) tests. Data is depicted as median ± interquartile ranges (IQR). Sholl analysis is shown as Mean ± standard error of the mean (SEM). *P* values < 0.05 were considered statistically significant (* = *p* < 0.05, ** = *p* < 0.01, *** = *p* < 0.001). All figures were prepared with Corel Draw X7 (Corel, Canada).

## Results

### NMDARs are involved in CT100-induced changes of synaptic function in young mice

Synaptic dysfunction, one of the earliest events in AD pathology [51], is thought to be caused by overproduction of toxic Aβ species [30, 95]. To induce Aβ-toxicity, we overexpressed Aβ in the DG of adult mice using a virus-mediated approach. To this end, we injected rAAVs that expressed the penultimate Aβ precursor CT100 (Additional file 1: S1a), which is known to reduce functional synapse number of neurons in organotypic slice cultures [67]. However, CT100 overexpression for three (data not shown, Table 1) and 9–10 weeks (Table 1 and Additional file 1: S1d) did not affect the number of functional synapses in adult mice, as mEPSC frequency in infected granule cells was unaffected compared to that in control cells. This was surprising because CT100 overexpression with a sindbis virus in organotypic slice cultures has been shown to induce synaptic dysfunction 24 h post infection [36, 67]. Since organotypic brain slices are prepared from newborn mice, we wondered if neurons in the brains of younger mice are more susceptible to Aβ-toxicity. We thus injected rAAVs overexpressing CT100 into the DG of young mice (P7) and indeed observed a decrease in mEPSC frequency in CT100-overexpressing cells 9–10 weeks post injection (Table 1 and Additional file 1: S1f), possibly suggesting that Aβ-toxicity reduces with brain development. However, infection efficacy and Aβ-overproduction may also change with development, which could explain the observed age-dependency.

**Table 1:** mEPSC recordings of CT100-overexpressing DG granule cells

Adult mice					
3w pi	Control (*n* = 22)		CT100 (*n* = 11)		
Frequency [Hz]	0.59 [0.37–0.77]		0.48 [0.44–0.71]		MW test: *p* = 0.9074
10w pi	Control (*n* = 40)		CT100 (n = 19)		
Frequency [Hz]	0.71 [0.4–0.92]		0.54 [0.42–0.77]		MW test *p* = 0.21
Young mice					
9w pi	Control (n = 56)		CT100 (n = 26)		
Frequency [Hz]	0.81 [0.51–1.02]		0.61 [0.41–0.81]		MW test: *p* = 0.047
GluN1^fl/fl^ 9w pi	Control (n = 34)	CT100 (n = 10)	GluN1^−/−^ (n = 21)	GluN1^−/−^ + CT100 (n= 9)	
Frequency [Hz]	0.69 [0.55–0.83]	0.37 [0.32–0.55]	0.99 [0.76–1.22]	1.18 [1.08–1.47]	Kruskal-Wallis: *p* < 0.0001; Dunn’s posttest: control vs CT100 *p* = 0.04; control vs GluN1^−/−^*p* = 0.0045; GluN1^−/−^ vs GluN1^−/−^ + CT100 *p* = 0.7895
Amplitude [pA]	10.47 [9.27–11.53]	10.47 [9.94–11.53]	14.85 [12.96–16.09]	12.4 [10.99–13.81]	Kruskal-Wallis: *p* < 0.0001; Dunn’s posttest: control vs CT100 *p* > 0.9999; control vs GluN1^−/−^*p* < 0.0001; GluN1^−/−^ vs GluN1^−/−^ + CT100 *p* = 0.336

To investigate the role of NMDARs in Aβ-mediated changes in synapse number and function, we deleted NMDARs subunits by injection of a Cre-recombinase expressing rAAV (rAAV-Syn-Cre-T2A-GFP) into the DG of mice, in which the gene encoding the GluN1 subunit (*grin1*) is flanked by *loxP* sites (GluN1^fl/fl^ mice). The ratio of NMDAR-mediated to AMPAR-mediated current amplitudes (NMDA/AMPA ratio) was significantly decreased (86.83%) three weeks post injection, indicating a nearly complete loss of NMDARs (Additional file 2: S4a, b and Table 2).

**Table 2:** NMDAR-mediated currents in 5xFAD DG granule cells and virus-infected cells

	Control (n = 16)	GluN1^−/−^ (n = 15)	
NMDAR/AMPAR ratio	1 ± 0.65	0.13 ± 0.04	MW test: p < 0.0001
	WT (n = 22)	5xFAD (n = 29)	
NMDAR/AMPAR ratio	1.18 [0.79–1.77]	0.72 [0.43–1.2]	MW test: *p* = 0.0029
	WT (n = 18)	5xFAD (n = 25)	
Decay tau [ms]	62.91 [57.75–67.48]	66.51 [59.2–72.86]	MW test: *p* = 0.0969
	WT (n = 23)	5xFAD (n = 22)	
Extrasynaptic amplitude [pA]	125.3 [85.8–178.6]	77.57 [43.12–101.2]	MW test: *p* = 0.0003
	WT (n = 23)	5xFAD (n = 22)	
Deactivation [ms]	74.76 [63.62–88.33]	79.43 [71.22–104.3]	MW test: *p* = 0.1712

In the next step, NMDARs were deleted in parallel with overexpression of Aβ to investigate whether NMDARs play a role in Aβ-mediated changes in synaptic function in young mice. To this end, one week old GluN1^fl/fl^ mice were injected with the following viruses: rAAV-CaMKII-tdTom (control cells = GluN1^fl/fl^), rAAV-CaMKII-CT100-T2A-tdTom (CT100 overexpression), rAAV-Syn-Cre-T2A-GFP (GluN1 deletion: GluN1^−/−^) and rAAV-Syn-Cre-T2A-GFP + rAAV-CaMKII-CT100-T2A-tdTom (GluN1^−/−^ and CT100 overexpression) (Additional file 1: S1a). Nine weeks after rAAV injection, mEPSCs were recorded from infected DG granule cells. In accordance with the results shown above, mEPSC frequency was reduced in CT100 overexpressing DG granule cells (Additional file 3: S2c, blue bar). Furthermore, an increase in mEPSC frequency and amplitude was observed in GluN1^−/−^ cells (red bar). Since deletion of GluN1 per se increased mEPSC frequency, we tested for an involvement of NMDARs in Aβ-toxicity by comparing GluN1^−/−^ cells with CT100/GluN1^−/−^ cells. Importantly, mEPSC frequency of CT100 expressing GluN1^−/−^ cells was not significantly different from the mEPSC frequency in GluN1^−/−^ cells (Additional file 3: S2c, grey bar and Table 1). This indicates that NMDARs mediate the Aβ-induced reduction in the number of functional synapses. However, we cannot exclude that NMDARs and Aβ affect functional synapse number via independent parallel pathways. While performing electrophysiological recordings, neurons were filled with biocytin to subsequently perform morphological analysis. Interestingly, spine density was increased in dendrites of Aβ-overexpressing DG granule cells with no change in the distribution of stubby, thin and mushroom spines (Additional file 3: S2d, g, Tables 3 and 4). Thus, unexpectedly, Aβ increased the number of spines while in parallel reducing the number of functional synapses.

**Table 3:** Morphological analysis of CT100-overexpressing DG granule cells

GluN1^fl/fl^ 9w pi					
Spine numbers	Control (n = 23)	CT100 (n = 6)	GluN1^−/−^(n= 23)	GluN1^−/−^ + CT100 (n = 10)	
	1.54 [1.25–1.84]	2.22 [1.95–2.37]	1.73 [1.54–1.9]	1.51 [1.34–1.62]	Kruskal-Wallis: *p* = 0.0015; Dunn’s posttest: control vs CT100 *p* = 0.0026; control vs GluN1^−/−^ *p* = 0.1332; GluN1^−/−^ vs GluN1^−/−^ + CT100 *p* = 0.1119

**Table 4:** Values for spine morphology in CT100 and CT100(I716F) overexpression experiments

	Spine morphology distribution [%]		
	Stubby	Thin	Mushroom
9w pi CT100 in P7 floxed GluN1			
Control (23)	0.29 [0.26–0.31]	0.62 [0.6–0.67]	0.09 [0.47–0.11]
CT100 (6)	0.36 [0.27–0.4]	0.59 [0.54–0.64]	0.06 [0.04–0.09]
GluN1^−/−^ (10)	0.29 [0.23–0.35]	0.6 [0.57–0.7]	0.07 [0.05–0.1]
GluN1^−/−^ + CT100 (23)	0.27 [0.23–0.32]	0.64 [0.62–0.67]	0.07 [0.05–0.11]
Kruskal Wallis test (Dunn’s posttest)	*P* = 0.1972 (Control vs CT100: *p* = 0.4813 Control vs GluN1−/: *p* > 0.9999; GluN1−/− vs GluN1−/− + CT100: *p* > 0.9999)	*P* = 0.1433 (Control vs CT100: *p* = 0.6288; Control vs GluN1^−/^: *p* > 0.9999; GluN1^−/−^ vs GluN1^−/−^ + CT100: *p* = 0.5863)	*P* = 0.8439 (Control vs CT100: *p* > 0.9999; Control vs GluN1^−/^: *p* > 0.9999; GluN1^−/−^ vs GluN1^−/−^ + CT100: *p* > 0.9999)
DG granule cells GluN1^−/−^ line			
Control (49)	0.32 [0.27–0.36]	0.61 [0.54–0.64]	0.08 [0.05–0.10]
CT100(I716F) (19)	0.29 [0.27–0.33]	0.63 [0.59–0.66]	0.08 [0.06–0.08]
GluN1^−/−^ (22)	0.32 [0.26–0.39]	0.56 [0.49–0.64]	0.1 [0.06–0.14]
GluN1^−/−^ + CT100(I716F) (28)	0.32 [0.27–0.37]	0.61 [0.55–0.67]	0.07 [0.05–0.85]
Kruskal Wallis test (Dunn’s posttest)	*p* = 0.529 (Control vs CT100(I716F): p = 0.53; Control vs GluN1^−/−^: *p* > 0.9999; GluN1^−/−^ vs GluN1^−/−^ + CT100(I716F): *p* > 0.9999)	*p* = 0.198 (Control vs CT100(I716F): *p* = 0.5339; Control vs GluN1^−/−^: *p* = 0.8345; GluN1^−/−^ vs GluN1^−/−^ + CT100(I716F): *p* = 0.3877)	*p* = 0.1098 (Control vs CT100(I716F): *p* > 0.9999; Control vs GluN1^−/−^: *p* = 0.2132; GluN1^−/−^ vs GluN1^−/−^ + CT100(I716F): *p* = 0.0511)
DG granule cells GluN2A^fl/fl^ line			
Control (11)	0.35 [0.32–0.37]	0.57 [0.53–0.62]	0.08 [0.03–0.11]
CT100(I716F) (17)	0.36 [0.29–0.38]	0.57 [0.52–0.62]	0.07 [0.06–0.11]
GluN2A^−/−^ (26)	0.38 [0.34–0.42]	0.54 [0.49–0.58]	0.1 [0.06–0.18]
GluN2A^−/−^ + CT100(I716F) (21)	0.34 [0.3–0.38]	0.55 [0.52–0.61]	0.1 [0.08–0.13]
Kruskal Wallis test (Dunn’s posttest)	*p* = 0.1208 (Control vs CT100(I716F): p > 0.999; Control vs GluN1^−/−^: *p* = 0.9455; GluN1^−/−^ vs GluN1^−/−^ + CT100(I716F): *p* = 0.0586)	*p* = 0.2321 (Control vs CT100(I716F): *p* > 0.9999; Control vs GluN1^−/−^: *p* = 0.2893; GluN1^−/−^ vs GluN1^−/−^ + CT100(I716F): *p* = 0.7813)	*p* > 0.1487 (Control vs CT100(I716F): *p* > 0.9999; Control vs GluN1^−/−^: *p* > 0.9999; GluN1^−/−^ vs GluN1^−/−^ + CT100(I716F): *p* = 0.4372)
DG granule cells GluN2B^fl/fl^ line			
Control (31)	0.36 [0.33–0.42]	0.55 [0.49–0.58]	0.08 [0.06–0.11]
CT100(I716F) (45)	0.32 [0.28–0.4]	0.59 [0.53–0.6442]	0.07 [0.05–0.1]
GluN2B^−/−^ (29)	0.33 [0.28–0.39]	0.56 [0.49–0.6]	0.11 [0.09–0.14]
GluN2B^−/−^ + CT100(I716F) (16)	0.37 [0.33–0.44]	0.57 [0.5–0.6]	0.07 [0.04–0.09]
Kruskal Wallis test (Dunn’s posttest)	*P* = 0.0105 (Control vs CT100(I716F): *p* = 0.0277; Control vs GluN2B^−/−^: *p* = 0.043; GluN2B^−/−^ vs GluN2B^−/−^ + CT100(I716F): *p* = 0.1063)	*P* = 0.0319 (Control vs CT100(I716F): *p* = 0.0112; Control vs GluN2B^−/−^: *p* = 0.6837; GluN2B^−/−^ vs GluN2B^−/−^ + CT100(I716F): *p* > 0.9999)	*P* = 0.0002 (Control vs CT100(I716F): p > 0.9999; Control vs GluN2B^−/−^: *p* = 0.0066; GluN2B^−/−^ vs GluN2B^−/−^ + CT100(I716F): *p* = 0.0048)

### CT100(I716F) overexpression reduces the number of functional synapses in adult mice

One intention of the study was to investigate the role of NMDARs for Aβ-toxicity in adult mice (12–16 weeks of age), i.e. at an age when the composition of NMDARs is similar to the composition in aging. However, CT100 overexpression was ineffective in adult mice (see above). We therefore generated a mutated version of CT100, in which isoleucine (I) at position 716 was exchanged to phenylalanine (F) (CT100(I716F)). This mutation alters the γ-secretase cleavage site and is known to increase the production of the toxic Aβ_42_ [28, 81]. Expression of CT100(I716F) in primary hippocampal cultures increased Aβ in the supernatant as verified by dot blot analysis (data not shown). Overexpression of CT100(I716F) induced a decrease in mEPSC frequency three weeks after rAAV injection into the DG of adult mice (Fig. 1e, h, k and Table 5). Thus, the increased production of Aβ_42_ resulting from the I716F mutation indeed affected synaptic function stronger than the unmutated CT100. To investigate whether the reduction in mEPSC frequency results from a decreased release probability, we investigated the paired pulse ratio (PPR) of medial perforant path synapses. CT100(I716F) did not affect the PPR (Fig. 1n and Table 6). This indicates that Aβ decreases mEPSC frequency by reducing synapse number or by increasing the number of synapses devoid of AMPAR (i.e silent synapses) [94]. Previous studies suggested that Aβ alters neuronal excitability [6, 90]. We therefore investigated intrinsic active and passive electrophysiological properties of DG granule cells (Additional file 4: S3). CT100(I716F) overexpression did not change threshold potential, action potential (AP) amplitude, duration, afterhyperpolarisation (AHP) and input resistance. Firing frequency, as well as early- and late adaptation were also not different between CT100(I716F) expressing DG granule cells and control cells (Additional file 4: S3b, c and Table 7). Thus, three-week overproduction of Aβ did not influence the active and passive properties of DG granule cells, but decreased the number of functional synapses.

**Table 5:** mEPSC recordings of CT100(I716F)-overexpressing DG granule cells

GluN1^fl/fl^	Control (n = 26)	CT100(I716F) (*n* = 33)	GluN1^−/−^ (*n* = 24)	GluN1^−/−^ + CT100(I716F) (*n* = 21)	
Frequency [Hz]	0.66 [0.52–0.77]	0.42 [0.3–0.64]	0.89 [0.69–1.63]	1.02 [0.68–1.23]	Kruskal-Wallis: p < 0.0001; Dunn’s posttest: control vs CT100(I716F) *p* = 0.029; control vs GluN1^−/−^ *p* = 0.044; GluN1^−/−^ vs GluN1^−/−^ + CT100(I716F) *p* > 0.99
Percentual reduction		0.36 [0.02–0.55]		−0.15 [− 0.38–0.23]	*p* = 0.0137
Amplitude [pA]	10.81 [10.03–11.47]	10.62 [10.02–11.13]	11.2 [10.34–12.44]	11.05 [9.78–12]	Kruskal-Wallis: *p* = 0.3076; Dunn’s posttest: control vs CT100(I716F) *p* > 0.99; control vs GluN1^−/−^ *p* = 0.75; GluN1^−/−^ vs GluN1^−/−^ + CT100(I716F) p > 0.99
GluN2A^fl/fl^	Control (n= 36)	CT100(I716F) (n= 37)	GluN2A^−/−^ (n= 24)	GluN2A^−/−^ + CT100(I716F) (n= 19)	
Frequency [Hz]	0.61 [0.5–0.75]	0.5 [0.31–0.68]	0.76 [0.6–0.92]	0.62 [0.46–0.85]	Kruskal-Wallis: *p* = 0.0004; Dunn’s posttest: control vs CT100(I716F) *p* = 0.0468; control vs GluN2A^−/−^ *p* = 0.149; GluN2A^−/−^ vs GluN2A^−/−^ + CT100(I716F) *p* = 0.485
Percentual reduction		0.19 [− 0.1–0.51]		0.18 [− 0.11–0.39]	*p* = 0.27
Amplitude [pA]	9.67 [8.46–10.32]	9.83 [9.3–10.82]	10.47 [9.29–12.96]	10.44 [9.84–11.95]	Kruskal-Wallis: p = 0.013; Dunn’s posttest: control vs CT100(I716F) *p* = 0.844; control vs GluN2A^−/−^ *p* = 0.75; GluN2A^−/−^ vs GluN2A^−/−^ + CT100(I716F) p > 0.99
GluN2B^fl/fl^	Control (n = 28)	CT100(I716F) (n= 25)	GluN2B^−/−^ (n = 27)	GluN2B^−/−^ + CT100(I716F) (n = 26)	
Frequency [Hz]	0.71 [0.53–1.08]	0.39 [0.28–0.75]	1.01 [0.81–1.23]	0.87 [0.72–1.03]	Kruskal-Wallis: p < 0.0001; Dunn’s posttest: control vs CT100(I716F) *p* = 0.013; control vs GluN2B^−/−^ *p* = 0.018; GluN2B^−/−^ vs GluN2B^−/−^ + CT100(I716F) *p* = 0.497
Percentual reduction		0.45 [− 0.06–0.6]		0.14 [− 0.02–0.29]	p = 0.1
Amplitude [pA]	9.58 [8.61–10.26]	9.65 [8.43–10.51]	9.8 [9.2–10.61]	11.59 [10.16–12.69]	Kruskal-Wallis: *p* < 0.0001; Dunn’s posttest: control vs CT100(I716F) *p* > 0.9999; control vs GluN2B^−/−^ *p* > .0.9999; GluN2B^−/−^ vs GluN2B^−/−^ + CT100(I716F) *p* = 0.0033

**Table 6:** Values of PPR of CT100(I716F)-overexpressing DG granule cells

	WT (n = 20)	CT100(I716F) (n = 20)	
25 ms ISI	0.84 [0.78–0.88]	0.81 [0.74–0.87]	MW-test: *p* = 0.2423
50 ms ISI	1.1 [1.05–1.17]	1.13 [1.07–1.26]	MW-test: *p* = 0.201

**Table 7:** Intrinsic and firing properties of CT100(I716F) overexpressing DG granule cells

	3w pi CT100(I716F)		
	Control	CT100(I716F)	
	n = 31	n = 20	
Passive properties			
Input resistance [mΩ]	182 [140–211.5]	170 [129.5–184]	MW test: *p* = 0.2418
Active properties			
AP threshold [mV]	−37.27 [−39.18 - -33.78]	−35.84 [− 39.04 - -30.2]	MW test: *p* = 0.5246
AP width [ms]	1.26 [1.2–1.32]	1.24 [1.15–1.28]	MW test: *p* = 0.3286
AP amplitude [mV]	94.03 [90.88–97.7]	91.25 [87.12–95.74]	MW test: p = 0.1308
AHP [mV]	−13.83 [−16–58- -10]	−13.76 [−15.77- - 11.23]	MW test: *p* = 0.7964
Firing properties			
Firing frequency [Hz]	22 [16–26]	20.5 [17.25–23.75]	MW test: *p* = 0.7484
Early adaptation [%]	451.7 [356–563.4]	391.4 [347.1–543.1]	MW test: *p* = 0.6064
Late adaptation [%]	41.98 [24.16–61.51]	42.37 [20.43–102.4]	MW test: *p* = 0.8231

### NMDARs are required for the Aβ-mediated reduction in functional synapse number in adult mice

We next asked whether NMDARs are involved in the Aβ-mediated changes in synapse function in adult mice. To this end, mEPSCs were recorded in three different mouse strains, each with conditional deletion of either GluN1, GluN2A, or GluN2B (GluN1^fl/fl^, GluN2A^fl/fl^ and GluN2B^fl/fl^). Conditional deletion of the subunits has been induced by injection of either one or two viruses: rAAV-CaMKII-tdTom (control cells = GluN1^fl/fl^, GluN2A^fl/fl^ or GluN2B^fl/fl^), rAAV-CaMKII-CT100(I716F)-T2A-tdTom (CT100(I716F) overexpression), rAAV-Syn-Cre-T2A-GFP (GluN1^−/−^ or GluN2A^−/−^ or GluN2B^−/−^ granule cells) and rAAV-Syn-Cre-T2A-GFP + rAAV-CaMKII-CT100(I716F)-T2A-tdTom (NMDAR subunit deletion together with CT100(I716F) overexpression) (Fig. 1a). Synaptic currents were recorded three weeks after virus injection. CT100(I716F) overexpression significantly decreased mEPSC frequency by 20–45% without affecting mEPSC amplitude (Fig. 1e, h, k, Additional file 2: S4d, f, h and Table 5). Deletion of the GluN1 subunit as well as deletion of the GluN2B subunit increased mEPSC frequency (Fig. 1e, n and Table 5) similar to the observations in young mice. Importantly, overexpression of CT100(I716F) in GluN1^−/−^, GluN2A^−/−^ and GluN2B^−/−^ granule cells did not significantly reduce mEPSC frequency (Fig. 1e, h, k and Table 5). Deletion of GluN1 abolished the effect of Aβ overproduction almost completely: the CT100(I716F)-mediated mEPSC frequency reduction in GluN1^−/−^ cells was significantly smaller than the reduction in wildtype cells (Fig. 1f). This indicates that the effect of Aβ on the number of functional synapses is mediated via NMDARs (since there are nearly no functional NMDARs in GluN1^−/−^ cells; Additional file 2: S4b and Table 2). The deletion of only GluN2A or GluN2B had a smaller impact on the effect of CT100(I716F) on mEPSC frequency (Fig. 1i, l).

**Fig. 2:**
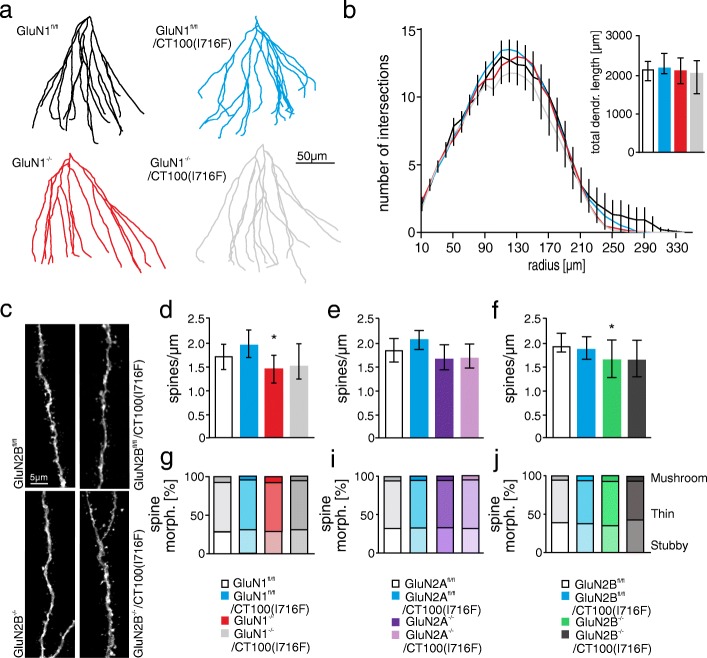
CT100(I716F) overexpression does not affect morphology of granule cells in adult mice. **a** Examples of traced DG granule cells after biocytin filling. **b** Sholl analysis shows that neither CT100(I716F)-overexpression nor GluN1 knockout affects the number of intersections of granule cell dendrites. There is no difference in the total dendritic length of neurons in the different groups. **c** Examples of maximum intensity projections of z-stacks of GluN2B^fl/fl^, GluN2B^fl/fl^/CT100(I716F), GluN2B^−/−^ and GluN2B^−/−^/CT100(I716F) granule cell dendrites. **d + e + f** Spine number is not affected after three weeks of CT100(I716F)-overexpression. Spine number is reduced in GluN1^−/−^ (**d**), and GluN2B^−/−^ (**f**) granule cells. **g + i + j** The distribution of stubby, thin and mushroom spines is slightly affected by CT100(I716F)-overexpression and/or NMDAR subunit knockout with fewer thin spines in GluN2B^fl/fl^ vs GluN2B^−/−^; GluN2B^−/−^ vs GluN2B^−/−^/CT100(I716F) and an increase in stubby spines in GluN2A^−/−^ vs GluN2A^−/−^/CT100(I716F); GluN2B^fl/fl^ vs GluN2B^fl/fl^/CT100(I716F); GluN2B^fl/fl^ vs GluN2B^−/−^. Bar graphs show median ± IQR. * = *p* < 0.05, ** = *p* < 0.01, *** = *p* < 0.001; morph. = morphology

CT100(I716F) overexpression and NMDAR subunit knockout did not affect total length and arborisation of granule cell dendrites except for subtle changes in dendritic complexity in CT100(I716F) overexpressing cells compared to GluN2B^−/−^ cells (Fig. 2a, b, Additional file 5: S5 and Table 8). Interestingly, Aβ overproduction via CT100(I716F) for 3 weeks did not influence the number of spines (Fig. 2c–f and Table 8) despite the Aβ-mediated reduction in functional synapse number (reduction in mEPSC frequency; Fig. 1e, h, k). This suggests that Aβ increases the number of silent synapses. The deletion of the GluN1 or GluN2B subunit reduced spine number (Fig. 2d, f and Table 8). Together with the increased mEPSC frequency (Fig. 1e, k), this indicates that deletion of GluN1 or GluN2B decreases silent synapse number. Spine morphology was not affected as shown by unaltered distributions of stubby, thin and mushroom spines in the GluN1^fl/fl^ and GluN2A^fl/fl^ line, but small changes were observed in the GluN2B^fl/fl^ line (Fig. 2g, i, j and Table 4).

**Table 8:** Morphology of CT100(I716F)-overexpressing DG granule cells

GluN1^fl/fl^					
Spine numbers	Control (*n* = 51)	CT100(I716F) (*n* = 20)	GluN1^−/−^ (*n* = 22)	GluN1^−/−^ + CT100(I716F) (*n* = 28)	
	1.7 [1.45–1.97]	1.96 [1.69–2.27]	1.46 [1.16–1.72]	1.51 [1.23–1.98]	Kruskal-Wallis: *p* = 0.0008; Dunn’s posttest: control vs CT100 *p* = 0.1308 control vs GluN1^−/−^ *p* = 0.0381; GluN1^−/−^ vs GluN1^−/−^ + CT100 p > 0.99
Total dendritic length [μm]	Control (*n* = 15)	CT100(I716F) (n = 27)	GluN1^−/−^ (*n* = 17)	GluN1^−/−^ + CT100(I716F) (*n* = 22)	
	2106 [1843–2325]	2155 [2018–2533]	2090 [1782–2418]	2019 [1495–2343]	Kruskal-Wallis: *p* = 0.0195; Dunn’s posttest: control vs CT100 *p* > 0.99 control vs GluN1^−/−^ p > 0.99; GluN1^−/−^ vs GluN1^−/−^ + CT100 p > 0.99
GluN2A^fl/fl^					
Spine numbers	Control (n = 11)	CT100(I716F) (n = 17)	GluN2A^−/−^(n = 26)	GluN2A^−/−^ + CT100(I716F) (n = 21)	
	1.81 [1.56–1.09]	2.97 [1.84–2.25]	1.65 [1.42–1.95]	1.67 [1.46–1.96]	Kruskal-Wallis: *p* = 0.0015; Dunn’s posttest: control vs CT100 *p* = 0.3366 control vs GluN2A^−/−^ *p* = 0.5077; GluN2A^−/−^ vs GluN2A^−/−^ + CT100 *p* > 0.9999
Total dendritic length [μm]	Control (n = 16)	CT100(I716F) (*n* = 17)	GluN2A^−/−^(n = 17)	GluN2A^−/−^ + CT100(I716F) (*n* = 18)	
	2162 [1657–2391]	1889 [1577–2155]	1862 [1528–2254]	2046 [1885–2189]	Kruskal-Wallis: *p* = 0.337; Dunn’s posttest: control vs CT100 *p* = 0.5642 control vs GluN2A^−/−^ *p* = 0.6218; GluN2A^−/−^ vs GluN2A^−/−^ + CT100 *p* = 0.6027
GluN2B^fl/fl^					
Spine numbers	Control (*n* = 31)	CT100(I716F) (*n* = 45)	GluN2B^−/−^(*n* = 29)	GluN2B^−/−^ + CT100(I716F) (*n* = 16)	
	1.91 [1.8–2.2]	1.88 [1.65–2.13]	1.63 [1.29–2.06]	1.55 [1.18–1.87]	Kruskal-Wallis: *p* = 0.0021; Dunn’s posttest: control vs CT100 *p* = 0.7884 control vs GluN2B^−/−^ *p* = 0.0151; GluN2B^−/−^ vs GluN2B^−/−^ + CT100 p > 0.99
Total dendritic length [μm]	Control (*n* = 29)	CT100(I716F) (n = 26)	GluN2B^−/−^(*n* = 23)	GluN2B^−/−^ + CT100(I716F) (n = 16)	
	2248 [2013–2577]	2223 [1882–2364]	2336 [2064–2681]	1882 [1668–2437]	Kruskal-Wallis: *p* = 0.1092; Dunn’s posttest: control vs CT100 *p* = 0.6198 control vs GluN2B^−/−^ *p* > 0.9999; GluN2B^−/−^ vs GluN2B^−/−^ + CT100 *p* = 0.1049

### NMDARs are not required for the spine loss in 5xFAD mice

**Fig. 3:**
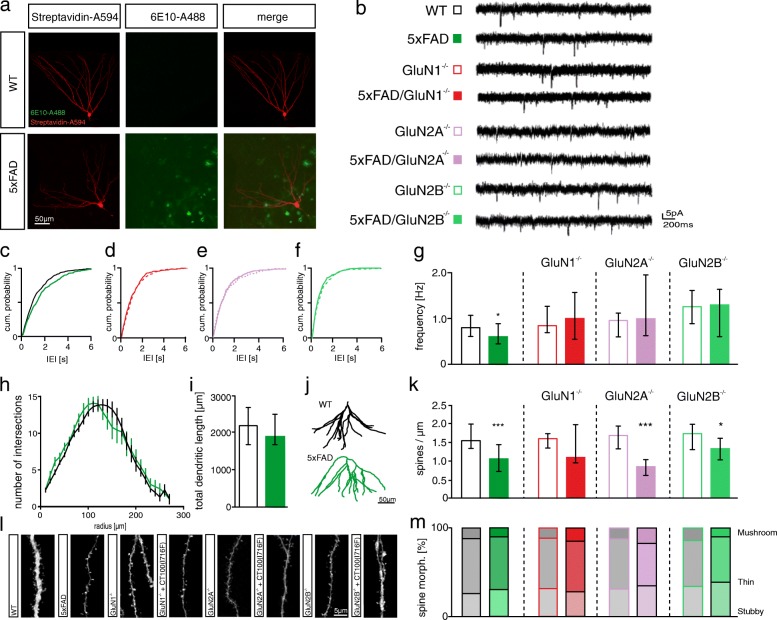
The synaptic depression in DG granule cells of 5xFAD mice is NMDAR dependent. **a** Biocytin filled granule cells (red) in brain slices of WT and 5xFAD mice. Aβ plaques in 5xFAD mice were visualized using a 6E10-coupled A488 antibody. No plaques are seen in WT mice. **b** Example traces of mEPSC recordings from granule cells of WT and 5xFAD mice with NMDAR subunit deletions. **c + d + e + f** Cumulative probability of the IEIs is shifted towards larger IEIs in cells of 5xFAD mice, but not in cells of 5xFAD/GluN1^−/−^, 5xFAD/GluN2A^−/−^ and 5xFAD/GluN2B^−/−^ mice**. g** mEPSC frequency is reduced in granule cells of 5xFAD mice. There is no difference in mEPSC frequency in granule cells of GluN1^−/−^ and 5xFAD/GluN1^−/−^, GluN2A^−/−^ and 5xFAD/GluN2A^−/−^ or GluN2B^−/−^ and 5xFAD/GluN2B^−/−^ mice. **h** The number of intersections is not changed in granule cells of 5xFAD mice. Number of intersections: Mean ± SEM. **i** Total dendritic length is not affected in granule cells of 5xFAD mice. **j** Examples of traced DG granule cells from one year old 5xFAD mice and WT littermates. **k S**pine number is decreased in granule cells of 5xFAD mice. There is a trend to a reduced spine numbers in 5xFAD/GluN1^−/−^ cells and a significantly decreased spine number in 5xFAD/GluN2A^−/−^ and 5xFAD/GluN2B^−/−^ granule cells. **l** Example images of maximum intensity projections of z-stacks from the different conditions analyzed for the spine counting. **m** Quantification of spine morphology distribution indicates that the decrease in spine number in DG granule cells of 5xFAD mice is not due to a loss of a specific spine subtype except for the 5xFAD/GluN2A^−/−^ cells, in which thin spines were reduced. Bar graphs show median ± IQR. * = *p* < 0.05, ** = *p* < 0.01, *** = *p* < 0.001; cum. = cumulative; morph. = morphology

Our data so far showed that Aβ-overproduction for three weeks decreases the number of functional synapses and that NMDARs are required for this effect. There was no decrease in spine number, which, however, is an early event in AD pathogenesis that correlates well with cognitive impairment [84]. The absence of a spine loss in CT100(I716F)-expressing cells may be explained by the relatively short time-period of CT100(I716F) expression and perhaps by a moderate Aβ overproduction using the virus-mediated approach. To analyze the role of NMDARs for Aβ-mediated spine loss, we thus employed 5xFAD mice, in which mutations in the *APP* and *PSEN1* genes result in the accumulation of high levels of Aβ_42_ [60]. In 12 months old 5xFAD mice, we detected Aβ plaques throughout the DG in close proximity to the investigated cells (Fig. 3a). Spine density and spine morphology was not changed in granule cells of six-month-old 5xFAD mice (Additional file 6: S6d, e and Table 9). Consistently, there was no change in mEPSC frequency, but we found an increase in mEPSC amplitude (Additional file 6: S6g and Table 10). In contrast, spine density and mEPSC frequency were significantly reduced in granule cells of one-year-old 5xFAD mice (Fig. 3g, k, Tables 9 and 10). Spine density reduction in 5xFAD mice was not due to loss of a specific morphological spine subtype (Fig. 3m and Table 11). Sholl analysis revealed no difference in dendritic arborization and total dendritic length between 5xFAD and WT mice (Fig. 3h, i and Table 9).

**Table 9:** Morphological analysis of the 5xFAD mouse model

6 m DG			
Spine numbers	WT (n = 17)	5xFAD (n = 20)	
	1.25 [0.93–1.56]	1.36 [1–1.6]	MW-test: *p* = 0.8923
Total dendritic length	WT (*n* = 13)	5xFAD (n = 20)	
	2707 [2131–3003]	2425 [2134–2630]	MW-test: *p* = 0.1275
1a DG			
Spine numbers	WT (n = 27)	5xFAD (n = 28)	
	1.54 [1.35–1.99]	1.07 [0.73–1.46]	MW-test: p < 0.0001
	GluN1^−/−^ (*n* = 6)	5xFAD/GluN1^−/−^ (*n* = 12)	
	1.62 [1.36–1.76]	1.13 [0.96–2.02]	MW-test: *p* = 0.325
	GluN2A^−/−^ (n = 12)	5xFAD/GluN2A^−/−^ (*n* = 10)	
	1.7 [1.36–1.98]	0.87 [0.64–1.04]	MW-test: *p* = 0.0001
	GluN2B^−/−^ (n = 28)	5xFAD/GluN2B^−/−^ (n = 13)	
	1.73 [1.29–1.96]	1.33 [1.02–1.6]	MW-test: *p* = 0.0165
Total dendritic length [μm]	WT (n = 22)	5xFAD (n = 19)	
	2222 [1704–2660]	1882 [1708–2480]	MW-test: *p* = 0.4763

**Table 10:** mEPSC recordings from the 5xFAD mouse model

6 m DG			
	WT (n = 24)	5xFAD (n = 23)	
Frequency [Hz]	0.73 [0.44–0.91]	0.66 [0.45–1.2]	MW-test: *p* = 0.6612
Amplitude [pA]	10.08 [9.15–10.52]	10.64 [10.15–11.7]	MW-test: *p* = 0.0013
1a DG			
	WT (n = 27)	5xFAD (n = 21)	
Frequency [Hz]	0.80 [0.61–1.07]	0.61 [0.44–0.89]	MW-test: *p* = 0.026
Amplitude [pA]	10.44 [8.54–11.95]	11.2 [8.94–11.86]	MW-test: *p* = 0.47
	GluN1^−/−^ (n = 17)	5xFAD/ GluN1^−/−^ (n = 17)	
Frequency [Hz]	0.85 [0.68–1.26]	1.02 [0.55–1.56]	MW-test: *p* = 0.8119
Amplitude [pA]	11.2 [10.24–12.84]	10.43 [9.66–12.31]	MW-test: *p* = 0.394
	GluN2A^−/−^ (n = 23)	5xFAD/ GluN2A^−/−^ (n = 17)	
Frequency [Hz]	0.97 [0.61–1.13]	1.01 [0.64–1.98]	MW-test: *p* = 0.2802
Amplitude [pA]	9.0 [8.38–9.93]	9.45 [8.97–10.72]	MW-test: *p* = 0.1626
	GluN2B^−/−^ (n = 21)	5xFAD/ GluN2B^−/−^ (n = 16)	
Frequency [Hz]	1.26 [0.89–1.61]	1.31 [0.6–1.63]	MW-test: p > 0.999
Amplitude [pA]	11.63 [10.72–12.14]	10.06 [9.88–12.92]	MW-test: *p* = 0.3232

**Table 11:** Overview of values for spine morphology in 5xFAD mice

	Spine Morphology distribution [%]		
	Stubby	Thin	Mushroom
6 m 5xFAD DG			
WT (17)	0.31 [0.25–0.34]	0.6 [0.56–0.64]	0.1 [0.06–0.1]
5xFAD (18)	0.32 [0.27–0.35]	0.58 [0.56–0.61]	0.1 [0.09–0.11]
Mann-Whitney test	*p* = 0.4	*p* = 0.142	*p* = 0.85
1a 5xFAD DG			
WT (29)	0.26 [0.22–0.35]	0.64 [0.6–0.7]	0.08 [0.04–0.11]
5xFAD (28)	0.32 [0.23–0.38]	0.59 [0.23–0.38]	0.09 [0.06–0.16]
Mann-Whitney test	*p* = 0.32	*p* = 0.13	*p* = 0.14
GluN1^−/−^ (7)	0.32 [0.27–0.39]	0.56 [0.45–0.62]	0.12 [0.12–0.16]
5xFAD/GluN21^−/−^ (12)	0.29 [0.25–0.31]	0.57 [0.47–0.63]	0.14 [0.12–0.21]
Mann-Whitney test	*p* = 0.16	*p* = 0.526	*p* = 0.29
GluN2A^−/−^ (12)	0.31 [0.24–0.36]	0.58 [0.47–0.64]	0.13 [0.09–0.17]
5xFAD/GluN2A^−/−^ (10)	0.39 [0.32–0.5]	0.39 [0.29–0.46]	0.19 [0.14–0.26]
Mann-Whitney test	*p* = 0.02	*p* = 0.002	*p* = 0.025
GluN2B^−/−^ (15)	0.35 [0.27–0.4]	0.53 [0.45–0.62]	0.1 [0.08–0.14]
5xFAD/GluN2B^−/−^ (11)	0.42 [0.34–0.52]	0.47 [0.37–0.54]	0.13 [0.1–0.16]
Mann-Whitney test	*p* = 0.0362	*p* = 0.0687	*p* = 0.3565

To investigate the role of NMDARs in Aβ-mediated synapse dysfunction and spine density reduction, 5xFAD mice were bred with conditional NMDAR KO lines (GluN1^fl/fl^, GluN2A^fl/fl^, and GluN2B^fl/fl^ mice). Cre-recombinase expressing rAAVs were injected into the DG of nine-month old 5xFAD/GluN1^fl/fl^, 5xFAD/GluN2A^fl/fl^ and 5xFAD/GluN2B^fl/fl^ mice and littermate controls (GluN1^fl/fl^, GluN2A^fl/fl^ and GluN2B^fl/fl^) to induce NMDAR subunit deletion (GluN1^−/−^, GluN2A^−/−^ and GluN2B^−/−^ cells). There was no difference in mEPSC frequency between GluN1^−/−^ and 5xFAD/GluN1^−/−^ granule cells (Fig. 3g, red bars and Table 10). Similarly, the mEPSC frequency was not different between GluN2A^−/−^ and 5xFAD/GluN2A^−/−^ granule cells as well as between GluN2B^−/−^ and 5xFAD/GluN2B^−/−^ granule cells (Fig. 3g, light green bars and Table 10). Thus, NMDARs are required for the reduction in the number of functional synapses in paradigms with short-time (with CT100(I716F) expression for three weeks) and chronic Aβ-overproduction (in 5xFAD mice). In fact, the protection that was induced by NMDAR subunit deletion was more evident in 5xFAD mice than in cells with CT100(I716F) expression. In addition, the deletion of only one subunit (GluN2A or GluN2B) was sufficient to abolish the influence of Aβ on functional synapse number in 5xFAD mice.

Knockout of GluN1, GluN2A or GluN2B per se did not affect spine density of granule cells. Importantly, deletion of GluN2A or GluN2B did not prevent the reduction in spine number in dendrites of granule cells in 5xFAD mice (Fig. 3k, light green bars and Table 9). The highly variable spine number in 5xFAD/GluN1^−/−^ cells reduces the informative value of the non-significant difference to the spine number in GluN1^−/−^ cells (Fig. 3k, red bars and Table 9). This hampers conclusions about the role of GluN1. However, the strong trend to reduced spine numbers in 5xFAD/GluN1^−/−^ cells indicates that NMDARs play a small role in the Aβ-mediated spine number reduction, in contrast to their requirement for the Aβ-mediated changes in functional synapse number.

### Aβ decreases surface expression of NMDARs

**Fig. 4:**
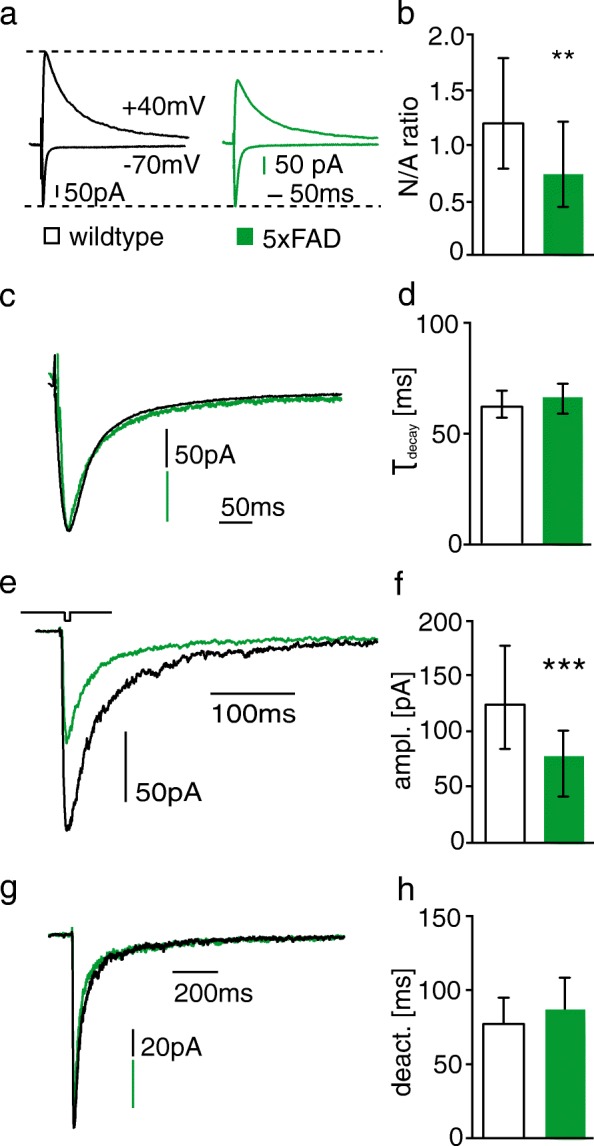
The amplitude of synaptic and extrasynaptic NMDAR-mediated currents is reduced in 5xFAD mice. **a** Example traces of NMDAR- and AMPAR-mediated currents recorded at holding potential of − 70 mV and + 40 mV, respectively, in DG granule cells of WT and 5xFAD mice. **b** The NMDAR/AMPAR (N/A) ratio is significantly reduced in DG granule cells of 5xFAD mice. **c** Example traces of NMDAR-mediated currents recorded at -30 mV. **d** The time constant of decay currents is not different between WT and 5xFAD cells. **e** Example traces of extrasynaptic NMDAR-mediated currents evoked by ultrafast-application of glutamate onto nucleated patches. **f** The peak amplitude of NMDAR-mediated currents is significantly reduced in granule cells of 5xFAD mice. **g** Example traces of normalized extrasynaptic NMDAR-mediated currents. **h** There is no difference in the deactivation time constant between DG granule cells of WT and 5xFAD mice. Bar graphs show median ± IQR. * = *p* < 0.05, ** = *p* < 0.01, *** = *p* < 0.001, ampl. = amplitude, deact. = deactivation

Results from the experiments described above showed that NDMARs are involved in Aβ-mediated changes of synapse function in adult mice. However, changes in the expression of NMDARs altered the number of functional synapses also in control mice. Thus, an important question is if Aβ reduces the number of functional synapses by influencing the surface expression of NMDARs. To address this question, we investigated synaptic and extrasynaptic NMDAR-mediated currents in granule cells of one-year-old 5xFAD mice. The NMDAR/AMPAR ratio was reduced in 5xFAD mice when compared to that in WT littermates (Fig. 4a, b and Table 2). This suggests that the number of synaptic NMDARs is markedly reduced in one-year-old 5xFAD mice considering the reduction in frequency of AMPAR-mediated mEPSCs (Fig. 3g). The gating kinetics of NMDARs depend on their subunit composition. For example, the deactivation time-constant of GluN1/GluN2A-containing NMDARs is 14 times smaller than that of GluN1/GluN2B-containing NMDARs [86]. Consequently, the deactivation time-constant of triheteromeric GluN1/GluN2A/GluN2B-containining NMDARs is with 78.7 ms in between that of the diheteromeric NMDARs [86]. Decay time constant of synaptic NMDAR-mediated currents was unaltered in one-year-old 5xFAD mice (Fig. 4c, d and Table 2). As the decay time constant of synaptic NMDAR-mediated currents is mainly determined by the deactivation time constant, this result suggests that the subunit composition of synaptic NMDARs was not changed in 5xFAD mice. It has been shown that extrasynaptic NMDARs play an important role for mediating neuron dysfunction and cell death in various brain diseases that are connected to over-activation of NMDARs (for review see [62]). We studied extrasynaptic NMDAR-mediated currents by ultra-fast application of glutamate onto nucleated patches of granule cells from one-year-old 5xFAD and WT mice. The amplitude of extrasynaptic NMDAR-mediated currents was decreased in 5xFAD mice (Fig. 4e, f and Table 2), showing that Aβ overexpression reduces also the number of extrasynaptic NMDARs. The deactivation time constant was not changed in granule cells of 5xFAD mice (Fig. 4g, h and Table 2), indicating that the subunit composition of extrasynaptic NMDARs is not affected by Aβ-overproduction.

## Discussion

The open-channel NMDAR blocker Memantine has been shown to improve cognitive abilities in moderate-to-severe AD patients [68, 100]. A series of studies using rodent neurons additionally suggested that NMDARs are involved in the pathophysiology of AD [74, 79]. Importantly, there is evidence that NMDARs mediate Aβ-induced changes in synaptic function and neuronal morphology [45, 70, 74, 99]. However, conclusions about the role of NMDARs in Aβ-toxicity were mostly drawn from studies using cultured neurons, which are relatively immature, and mostly by using pharmacological tools. Thus, these studies do not allow drawing unequivocal conclusions about the contribution of NMDAR subunits to Aβ toxicity.

We show in this study that NMDARs are required for the Aβ-induced reduction of functional synapse number in adult mice. Thus, deletion of either subunit was sufficient to protect granule cells from loss of functional synapses in 5XFAD mice. Interestingly, deletion of GluN2A was effective in 1 year old 5XFAD mice, but did not prevent reduction of functional synapse number in CT100(I716F)-expressing cells of 4–5 months old mice. Perhaps the role of GluN2A for Aβ toxicity was different between 4 and 5 months and 1 year old mice because of an aging dependent upregulation of GluN2A. Age dependent changes in subunit expression may also explain why previous studies had shown that block of NMDARs with non-selective or GluN2B-specific antagonists, but not GluN2A-preferring antagonists prevent the Aβ-mediated depression of synaptic current amplitudes in organotypic slice cultures [37].

Virus-mediated deletion of NMDARs per se increases the number of functional synapses in DG granule cells as evidenced by the increase in mEPSC frequency. This was most pronounced in cells with deletion of the GluN2B subunit for three months. This is in line with previous studies on the influence of NMDARs on functional synapse number of cortical, CA1 and CA3 neurons [1, 19, 22, 96] and suggests that the GluN2B-mediated reduction of functional synapse number is a widespread homeostatic plasticity mechanism that controls the strength of neuronal communication in different neuron types. The underlying mechanism for the increase in the number of functional synapses is most likely an NMDAR-mediated Ca^2+^-influx, which activates intracellular signaling molecules such as such as PKA and CaMKII leading to an upregulation of AMPAR number on the cell surface and in synapses [32, 76]. GluN2B-containing NMDARs are not only involved in the homeostatic control of functional synapse number, but also in the activity-dependent long-term plasticity of synaptic strength in mature neurons. Thus, genetic deletion of the GluN2B subunit reduces synaptic LTP [93]. Interestingly, GluN2B-containing NMDARs are required for the Aβ-induced reduction of synaptic long-term potentiation [70].

Our experiments with adult 5xFAD mice showed that GluN2A and GluN2B are dispensable for the Aβ-induced spine reduction. This is in line with some studies showing that block of GluN2B-containing NMDARs does not prevent Aβ-mediated spine loss [23, 82], but contrasts the finding of others that the GluN2B subunit mediates spines loss [82]. However, a general role of NMDARs in Aβ-induced spine reduction was found in other studies [4, 66, 74, 99]. Differences in in the maturity of neurons may well account for the contrasting findings of these studies and our study. NMDARs contributed to the Aβ-induced spine reduction in immature cultured neurons (studies mentioned above), but not in the brain of adult mice as shown in our study. The impact of NMDARs on neuron morphology in general may decrease with development. The morphology of immature cultured neurons is more flexible and spine stability lower than in mature neurons of the adult brain, suggesting that the mechanisms that control neuron morphology and spine density may well differ between immature and mature neurons. Indeed, block of NMDARs with APV for 14 days decreases spine density in cultured neurons [10]. Genetic deletion of the GluN2B subunit during developmental stages at which the rate of changes in neuron morphology is still high results in reduced spine number and alterations in dendrite arborization of CA1, CA3 and DG granule cells [2, 15, 17, 19]. Similarly, we observed a lower number of spines three weeks after deletion of the GluN2B subunit in 3–4 months old mice. However, after chronic GluN2B deletion for three months in one year old mice this effect vanished. This may indicate that the influence of GluN2B decreases with brain age or alternatively that the absence of GluN2B function for longer a time-period is compensated by other mechanisms that influence spine density such as BDNF-signaling [46, 69], or activation of voltage-gated-Ca-channels [80].

Differences in the spine stability may also explain that Aβ overproduction for three weeks with a virus-mediated approach did not reduce spine density in adult mice (this study), but decreased spine density in organotypic slice cultures already after 2–7 days [30, 99]. However, there are several other possible differences between young and adult brains that may account for the age-dependent decrease in Aβ-toxicity, such as differences in virus-infection efficacy or Aβ-expression levels in infected neurons. The bigger effect of Aβ overproduction on spine density or synaptic function in some studies using organotypic slices of young mice than in our study with adult mice may also be explained by the different types of viruses that were used. Thus, Aβ overproduction may be more pronounced when using Sindbis viruses [37, 99] than when using rAAVs (this study) for CT100 overexpression. In addition, the mode of application determines extent and velocity of Aβ-toxicity. Thus, repeated Aβ-application into the DG of 1-year-old mice over 6 days is neurotoxic [5]. It is likely that peak Aβ concentration is higher in this approach than in brains with 10-weeks virus-mediated CT100 overexpression or in 6 months-old 5xFAD mice. We did observe a spine number reduction in granule cells of 12-months old 5xFAD mice. Again, the mechanisms that reduce spine number may differ between the direct application of high doses of Aß and the more chronic Aβ overproduction in 5xFAD mice, which might better resemble the pathological situation in the brain of AD patients. Differences in the mechanisms of Aβ-toxicity between immature and mature neurons and/or high and lower Aβ-concentration could explain that GluN2B-containing NMDARs are required for the Aβ-mediated spine reduction in cultured neurons [30, 75, 99], but not in 5xFAD mice. In conclusion, other studies and our findings suggest that pathophysiological mechanisms of Aβ-toxicity change with brain maturation. Of note, a possible higher Aβ-toxicity in immature than in adult brains is not at odds with the fact that AD is a disease of elderly people. Thus, Aβ concentration may increase with age. In addition, chronically elevated Aβ levels may be necessary to induce toxicity leading to AD.

Our results so far lead to the question: What is the possible link of Aβ and NMDARs? Aβ may alter NMDAR activity by different mechanisms: 1. direct interaction with NMDARs [11], 2. increased ambient glutamate levels (due to reduced glutamate reuptake) [45], or by changes in NMDAR expression [79]. Direct binding of Aß to or next to NMDARs influences their function of localization [13, 40]. For example, Aß has been shown to directly activate recombinant GluN2A- and GluN2B-containing NMDARs expressed in Xenopus oocytes [85]. An augmented and potentially toxic calcium influx may be the consequence from the direct Aβ with NMDAR interaction or increased ambient glutamate levels [3]. Interestingly, this effect is subunit-dependent in cultured cortical neurons: Activation of GluN2B-containing NMDARs elevates, whereas activation of GluN2A-containing NMDARs reduces intracellular calcium levels upon stimulation with Aß [16]. Interestingly, there is also evidence that activation of NMDARs by Aß may not require ion-flux via the channel pore suggesting a metabotropic function of NMDARs when activated by Aß [4, 37]. There are several proposed mechanisms by which Aβ may affect the expression of NMDARs on the cell surface. For example, Aβ reduces the expression of synaptic NMDARs in cultured neurons and Tg2576 mice possibly by activation of α-7 nicotinic receptors, which promotes receptor internalization in a PP2B and STEP-dependent fashion [38, 79]*.* Another mechanism may be the Aβ-mediated depletion of EphB2, which has been shown to reduce surface expression of NMDARs on DG granule cells [9]. Consistently, the current amplitude of synaptic NMDAR-mediated currents is reduced in DG granule cells of adult 5xFAD mice (this study) and CA1 neurons in organotypic slices that were infected with CT100-expressing viruses [37]. In contrast to the study of Kessels and colleagues, in which CT100 reduced preferentially the current amplitude of GluN2B-containing NMDARs, we did not find any indication for changes in the subunit composition [37, 67]. This difference may be well explained by a smaller contribution of GluN2B-containing NMDARs to synaptic currents in mature neurons than in immature neurons of organotypic slices. It has been hypothesized that the reduction in synaptic NMDAR number results not only from increased receptor internalization, but also from redistribution from synaptic to extrasynaptic sites [79]. A redistribution of NMDARs may contribute to Aβ-toxicity as the activation of synaptic NMDARs is thought to stimulate pro-survival signaling in neurons, whereas that of extrasynaptic NMDARs induces neuron apoptosis [25, 44], but see also: [101]. In fact, a redistribution of NMDARs is thought to play a role for the pathophysiology of another neurodegenerative disease, e.g. in Huntington’s disease. Thus, exposure of neurons to huntingtin decreases the expression of synaptic NMDARs [53, 61] and increases the expression of extrasynaptic GluN2B-containing NMDARs [53]. We analyzed currents mediated by extrasynaptic NMDARs to investigate if a similar receptor redistribution is also involved in AD. Interestingly, we observed a reduction in the amplitude of extrasynaptic NMDAR-mediated currents again without indication for changes in the subunit composition. This rules out that the toxic influence of Aβ results from a redistribution of NMDARs from synaptic to extrasynaptic sites or from a change in the composition of extrasynaptic NMDARs with a relative increase in GluN2B-containing NMDARs. In fact, the decay time constant of synaptic NMDAR-mediated currents was in a similar range to the time constant of extrasynaptic NMDAR-mediated currents (62 ms and 76 ms, respectively), suggesting that the composition of synaptic and extrasynaptic NMDARs is very similar.

Our observation that synaptic and extrasynaptic NMDAR-mediated current amplitudes reduced to a similar extent without changes in subunit composition is in accordance with findings from a post-mortem study of the brain of human AD patients and healthy controls. In this study the authors revealed a comparable downregulation of the GluN2A and GluN2B subunit in hippocampus, temporal and cingulate cortex [34]. However, other studies were indicative for a downregulation in the expression of preferentially GluN2B-containing NMDARs in the hippocampus of AD patients [54]. The fact that we did not observe changes in spine number three months after genetic deletion of the GluN2B and GluN1 subunit makes it is unlikely that the downregulation of synaptic or extrasynaptic NMDARs is responsible for the Aβ-mediated reduction in functional synapses and spine number. It is rather the activation of the remaining NMDARs that contributes to the Aβ-mediated changes in functional synapse number and NMDAR-independent mechanisms that mediate the spine loss of granule cells in adult mice. The NMDAR downregulation may therefore even reduce the effect of Aβ on functional synapse number.

## Conclusion

Using conditional NMDAR subunit KO mice, we showed that NMDARs are required for the influence of Aβ on the number of functional synapses of dentate gyrus granule cells. However, they were not responsible for the reduction in spine number that are observed after chronic Aβ-overproduction. Similar observations were made in somatosensory neurons (data not shown), indicating that the role of NMDARs in Aβ-toxicity is not specific for the dentate gyrus. Our data suggest that pharmacological block of NMDARs may reduce the influence of Aβ on synaptic function at early AD stages, but most likely does not prevent the changes in neuron morphology that are seen at later AD stages. This could also explain why the low affinity NMDAR antagonist Memantine alleviates cognitive symptoms to some extent, but does not halt or reverse the progression of AD [20, 48].

## Additional files

Additional file 1: **S1.** AAV-CT100 overexpression leads to synaptic depression in young mice. **a** pAAV constructs used for control conditions (tdTomato) and for stable co-expression of a fluorescent marker and CT100 (tdTomato) or Cre-recombinase (GFP). **b** Example traces of mEPSC recordings from adult and young control or CT100-overexpressing DG granule cells. **c + d** CT100 overexpression for 9 weeks does not reduce mEPSC frequency and does not change the cumulative propability of inter-event-intervals (IEIs) in DG granule cells from adult mice. **e + f** CT100 overexpression for 9 weeks reduces mEPSC frequency in DG granule cells from younger mice (injected at P7). Bar graphs show median ± IQR. * = *p* < 0.05, ** = *p* < 0.01, *** = *p* < 0.001 (PDF 1550 kb)

Additional file 2: **S4.** NMDAR subunit deletion does not influence mEPSC peak amplitude in DG granule cells. **a** Example traces of NMDAR/AMPAR (N/A) ratio recordings three weeks after injection of AAV-Cre-T2A-GFP. **b** N/A ratio is strongly reduced three weeks after NMDAR deletion (GluN1^−/−^) in comparison to cells injected with a control virus (AAV-T2A-tdTom = GluN1^fl/fl^). **c-h** CT100(I716F) overexpression does not influence peak amplitude (blue bars). Peak amplitude is increased in GluN2B^−/−^ compared to GluN2B^−/−^/CT100(I716F) DG granule cells. Bar graphs show median ± IQR. * = *p* < 0.05, ** = *p* < 0.01, *** = *p* < 0.001, norm. = normalized, cum. = cumulative, ampl. = amplitude (PDF 1391 kb)

Additional file 3: **S2.** Synaptic depression induced by CT100 overexpression is NMDAR dependent in young mice. **a** Example traces of mEPSC recordings from mice injected with AAV-Tom (GluN1^fl/fl^), AAV-CT100-T2A-Tom (GluN1^fl/fl^/CT100), AAV-Cre-T2A-GFP (GluN1^−/−^) or co-injected with AAV-CT100-T2A-Tom and AAV-Cre-T2A-GFP (GluN1^−/−^/CT100). **b** Cumulative probability of inter-event-interval (IEI) is shifted to longer IEIs in CT100(I716F) overexpressing cells. **c** mEPSC frequency is reduced in CT100-overexpressing and increased in GluN1^−/−^ DG granule cells. There is no difference between GluN1^−/−^ cells and GluN1^−/−^/CT100 DG granule cells. **e + f** Peak amplitude is increased in GluN1^−/−^ cells compared to GluN1^fl/fl^ cells. Cumulative probability of the amplitude is shifted towards larger amplitues in GluN1^−/−^ neuons. **d** CT100 increased the spine number of DG granule cells from slices of young mice. **g** The quantification of the spine morphology distribution shows no significant difference between the groups. Bar graphs show median ± IQR. * = *p* < 0.05, ** = *p* < 0.01, *** = *p* < 0.001; cum. = cumulative; morph. = morphology (PDF 1485 kb)

Additional file 4: **S3.** Active and passive properties of DG granule cells are not altered by CT100(I716F) overexpression. **a** Example traces of action potentials (APs) from control and CT100(I716F)-overexpressing DG granule cells. **b** CT100(I716F) overexpression does not alter the intrinsic properties threshold, amplitude, half-amplitude (HA) duration, afterhyperpolarization (AHP) and input resistance of DG granule cells compared to control cells. **c** Firing frequency, early- and late adaptation do not differ between control and CT100(I716F)-overexpressing DG granule cells. **d** Example traces of firing patterns of control and CT100(I716F) DG granule cells. Bar graphs show median ± IQR. (PDF 146 kb)

Additional file 5: **S5.** CT100(I716F) overexpression does not influence total dendritic length in adult mice. **a** Examples of traced DG granule cells of the GluN2A^fl/fl^ mouse line. **b** The number of intersections analyzed by Sholl analysis is not changed by CT100(I716F) overexpression, GluN2A subunit deletion and GluN2A deletion in combination with CT100(I716F) overexpression. Mean ± SEM. Total dendritic length is not different between the groups. **c** Examples of traced DG granule cells of the GluN2B^fl/fl^ mouse line. **d** Sholl analysis of the number of intersections shows subtle changes in dendritic complexity in GluN2B^−/−^/CT100(I716F) cells compared to their respective control (GluN2B^−/−^). Mean ± SEM. Total dendritic length is not different between the groups. Bar graphs show median ± IQR.; dendr. = dendritic, morph. = morphology (PDF 133 kb)

Additional file 6: **S6.** Functional and structural properties are not affected in six-month old 5xFAD mice. **a** Examples of traced DG granule cells of six-month old WT and 5xFAD mice. **b** The number of intersections per radius is not changed as revealed by a Sholl analysis of cells from 5xFAD and WT mice. Mean ± SEM. **c** Total dendritic length is also not changed. **d + e** Spine number and spine morphology is not affected in DG granule cells of 5xFAD compared to WT mice. **f** mEPSC example traces of WT and 5xFAD granule cells. **g + h** mEPSC frequency is not changed in 5xFAD compared to WT granule cells, but peak amplitude is increased. Bar graphs show median ± IQR. * = *p* < 0.05, ** = p < 0.01, *** = p < 0.001; dendr. = dendritic, morph. = morphology (PDF 100 kb)

Additional file 7: **Table S1.** mEPSC recordings of CT100-overexpressing DG granule cells. **Table S2.** Morphological analysis of CT100-overexpressing DG granule cells. **Table S3.** mEPSC recordings of CT100(I716F)-overexpressing DG granule cells. **Table S4.** Morphology of CT100(I716F)-overexpressing DG granule cells. **Table S5.** Values of PPR of CT100(I716F)-overexpressing DG granule cells. **Table S6.** mEPSC recordings from the 5xFAD mouse model. **Table S7.** Morphological analysis of the 5xFAD mouse model. **Table S8.** Intrinsic and firing properties of CT100(I716F) overexpressing DG granule cells. **Table S9.** NMDAR-mediated currents in 5xFAD DG granule cells and virus-infected cells. **Table S10.** Values for spine morphology in CT100 and CT100(I716F) overexpression experiments. **Table S11.** Overview of values for spine morphology in 5xFAD mice. (DOCX 49 kb)

## Abbreviations

ACSF: Artificial cerebral spine fluid
AD: Alzheimer Disease
AHP: Afterhyperpolarisation
AMPAR: α-amino-3-hydroxy-5-methyl-4-isoxazolepropionic acid receptor
AP: Action potential
Aβ: Amyloid Beta
CT100: C-terminal 100
DG: Dentate gyrus
LTP: Long-term-potentiation
mEPSC: miniature excitatory post-synaptic current
NMDA/AMPA ratio: NMDAR-mediated to AMPAR-mediated current amplitude ratio
NMDARs: *N*-methyl-D-aspartate receptors
PPR: Paired-pulse ratio
rAAVs: Recombinant adeno-associated viruses
SD: Standard deviation
