# Supplementary figures and images for: NMDA receptors mediate synaptic depression, but not spine loss in the dentate gyrus of adult amyloid Beta (Aβ) overexpressing mice
Source: Acta Neuropathol Commun. 2018 Oct 23;6:110. doi: 10.1186/s40478-018-0611-4 (PMC6198500; doi:10.1186/s40478-018-0611-4)

a

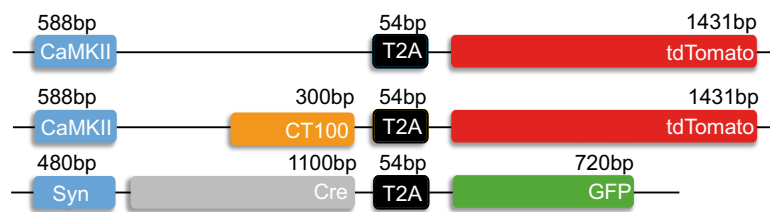

b

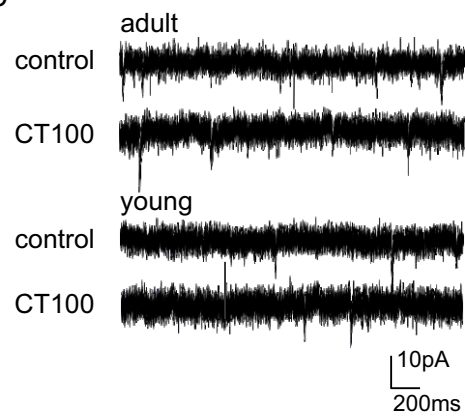

c

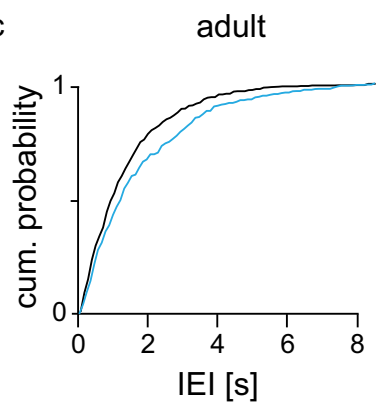

d

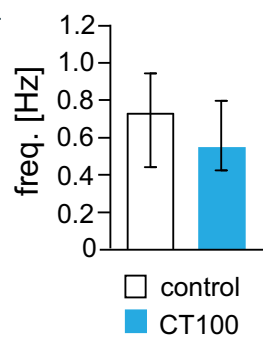

e

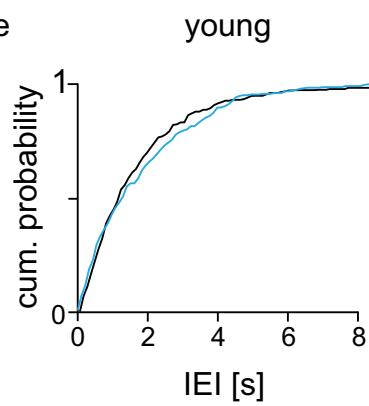

f

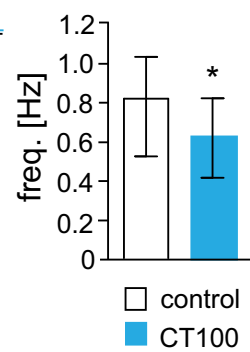

Supplement: Supplementary file 1 — S1. AAV-CT100 overexpression leads to synaptic depression in young mice. a pAAV constructs used for control conditions (tdTomato) and for stable co-expression of a fluorescent marker and CT100 (tdTomato) or Cre-recombinase (GFP). b Example traces of mEPSC recordings from adult and young control or CT100-overexpressing DG granule cells. c + d CT100 overexpression for 9 weeks does not reduce mEPSC frequency and does not change the cumulative propability of inter-event-intervals (IEIs) in DG granule cells from adult mice. e + f CT100 overexpression for 9 weeks reduces mEPSC frequency in DG granule cells from younger mice (injected at P7). Bar graphs show median ± IQR. * = p < 0.05, ** = p < 0.01, *** = p < 0.001 (PDF 1550 kb) [file 40478_2018_611_MOESM1_ESM.pdf]

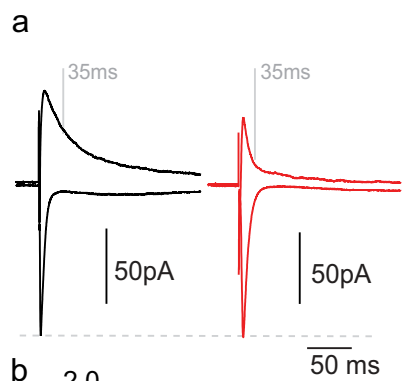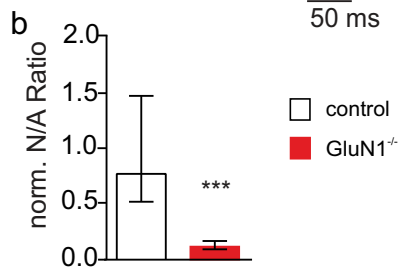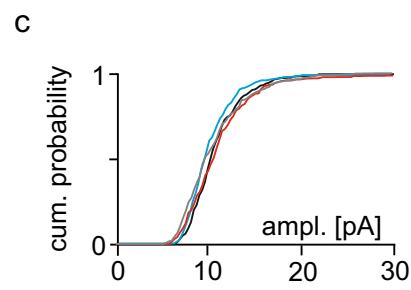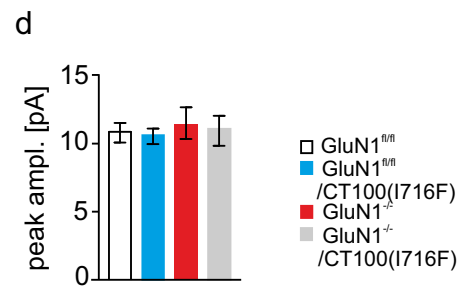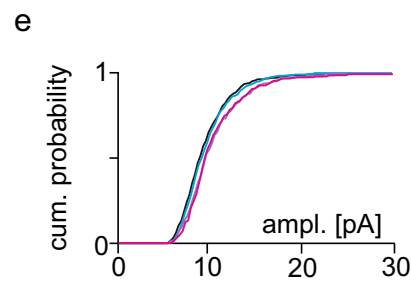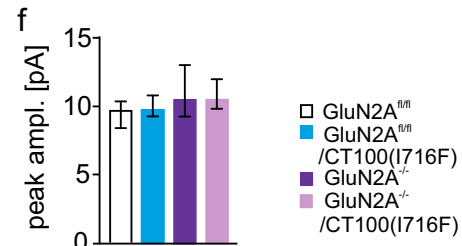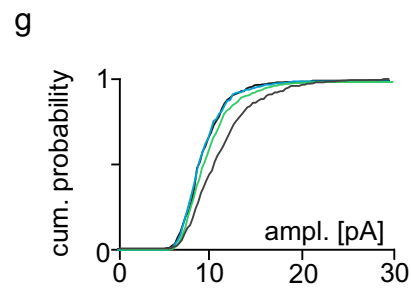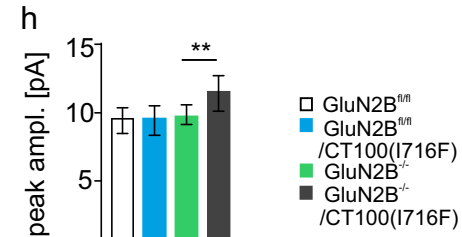

Supplement: Supplementary file 2 — S4. NMDAR subunit deletion does not influence mEPSC peak amplitude in DG granule cells. a Example traces of NMDAR/AMPAR (N/A) ratio recordings three weeks after injection of AAV-Cre-T2A-GFP. b N/A ratio is strongly reduced three weeks after NMDAR deletion (GluN1−/−) in comparison to cells injected with a control virus (AAV-T2A-tdTom = GluN1fl/fl). c-h CT100(I716F) overexpression does not influence peak amplitude (blue bars). Peak amplitude is increased in GluN2B−/− compared to GluN2B−/−/CT100(I716F) DG granule cells. Bar graphs show median ± IQR. * = p < 0.05, ** = p < 0.01, *** = p < 0.001, norm. = normalized, cum. = cumulative, ampl. = amplitude (PDF 1391 kb) [file 40478_2018_611_MOESM2_ESM.pdf]

a

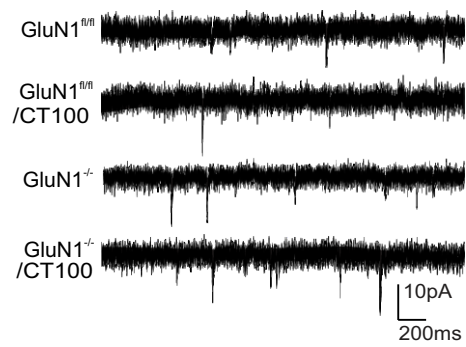

□  $\text{GluN1}^{\text{fl/fl}}$     ■  $\text{GluN1}^{\text{fl/fl}/\text{CT100}}$   
 ■  $\text{GluN1}^{-/-}$     ■  $\text{GluN1}^{-/-}/\text{CT100}$

b

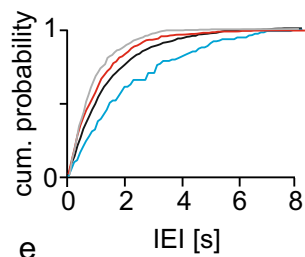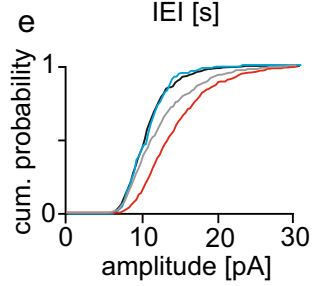

c

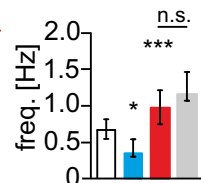

d

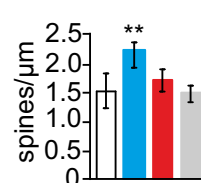

f

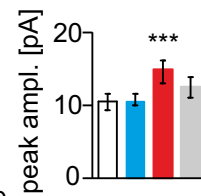

g

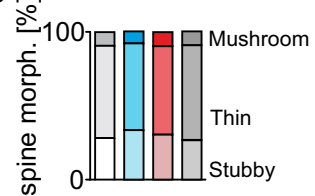

Supplement: Supplementary file 3 — S2. Synaptic depression induced by CT100 overexpression is NMDAR dependent in young mice. a Example traces of mEPSC recordings from mice injected with AAV-Tom (GluN1fl/fl), AAV-CT100-T2A-Tom (GluN1fl/fl/CT100), AAV-Cre-T2A-GFP (GluN1−/−) or co-injected with AAV-CT100-T2A-Tom and AAV-Cre-T2A-GFP (GluN1−/−/CT100). b Cumulative probability of inter-event-interval (IEI) is shifted to longer IEIs in CT100(I716F) overexpressing cells. c mEPSC frequency is reduced in CT100-overexpressing and increased in GluN1−/− DG granule cells. There is no difference between GluN1−/− cells and GluN1−/−/CT100 DG granule cells. e + f Peak amplitude is increased in GluN1−/− cells compared to GluN1fl/fl cells. Cumulative probability of the amplitude is shifted towards larger amplitues in GluN1−/− neuons. d CT100 increased the spine number of DG granule cells from slices of young mice. g The quantification of the spine morphology distribution shows no significant difference between the groups. Bar graphs show median ± IQR. * = p < 0.05, ** = p < 0.01, *** = p < 0.001; cum. = cumulative; morph. = morphology (PDF 1485 kb) [file 40478_2018_611_MOESM3_ESM.pdf]

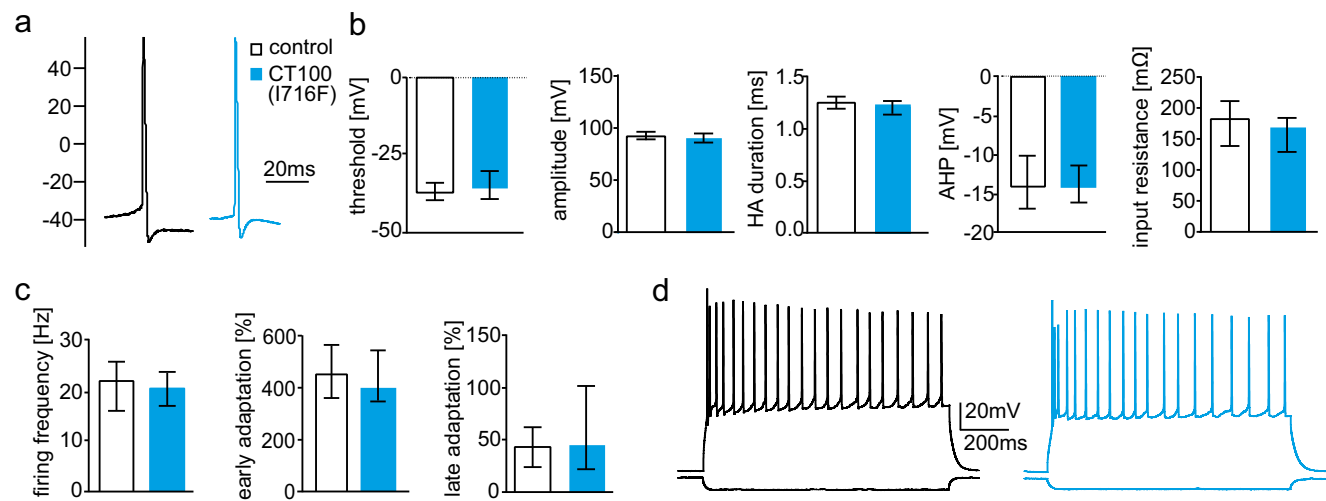

Supplement: Supplementary file 4 — S3. Active and passive properties of DG granule cells are not altered by CT100(I716F) overexpression. a Example traces of action potentials (APs) from control and CT100(I716F)-overexpressing DG granule cells. b CT100(I716F) overexpression does not alter the intrinsic properties threshold, amplitude, half-amplitude (HA) duration, afterhyperpolarization (AHP) and input resistance of DG granule cells compared to control cells. c Firing frequency, early- and late adaptation do not differ between control and CT100(I716F)-overexpressing DG granule cells. d Example traces of firing patterns of control and CT100(I716F) DG granule cells. Bar graphs show median ± IQR. (PDF 146 kb) [file 40478_2018_611_MOESM4_ESM.pdf]

a

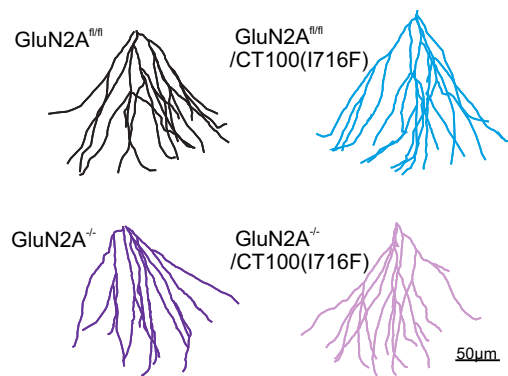

b

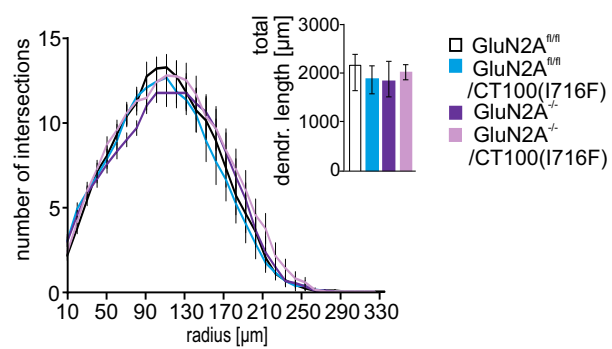

c

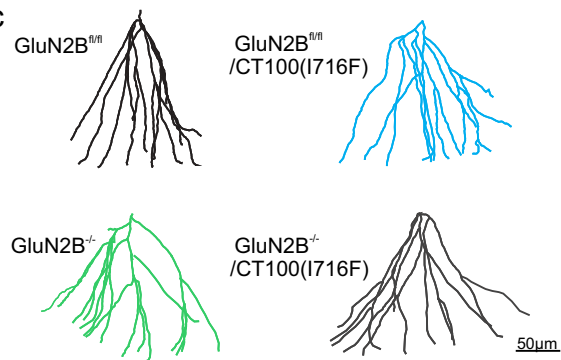

d

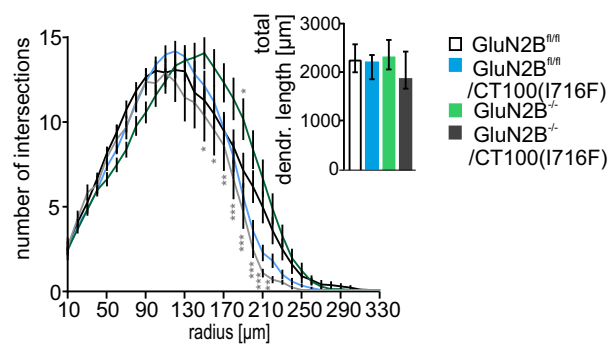

Supplement: Supplementary file 5 — S5. CT100(I716F) overexpression does not influence total dendritic length in adult mice. a Examples of traced DG granule cells of the GluN2Afl/fl mouse line. b The number of intersections analyzed by Sholl analysis is not changed by CT100(I716F) overexpression, GluN2A subunit deletion and GluN2A deletion in combination with CT100(I716F) overexpression. Mean ± SEM. Total dendritic length is not different between the groups. c Examples of traced DG granule cells of the GluN2Bfl/fl mouse line. d Sholl analysis of the number of intersections shows subtle changes in dendritic complexity in GluN2B−/−/CT100(I716F) cells compared to their respective control (GluN2B−/−). Mean ± SEM. Total dendritic length is not different between the groups. Bar graphs show median ± IQR.; dendr. = dendritic, morph. = morphology (PDF 133 kb) [file 40478_2018_611_MOESM5_ESM.pdf]

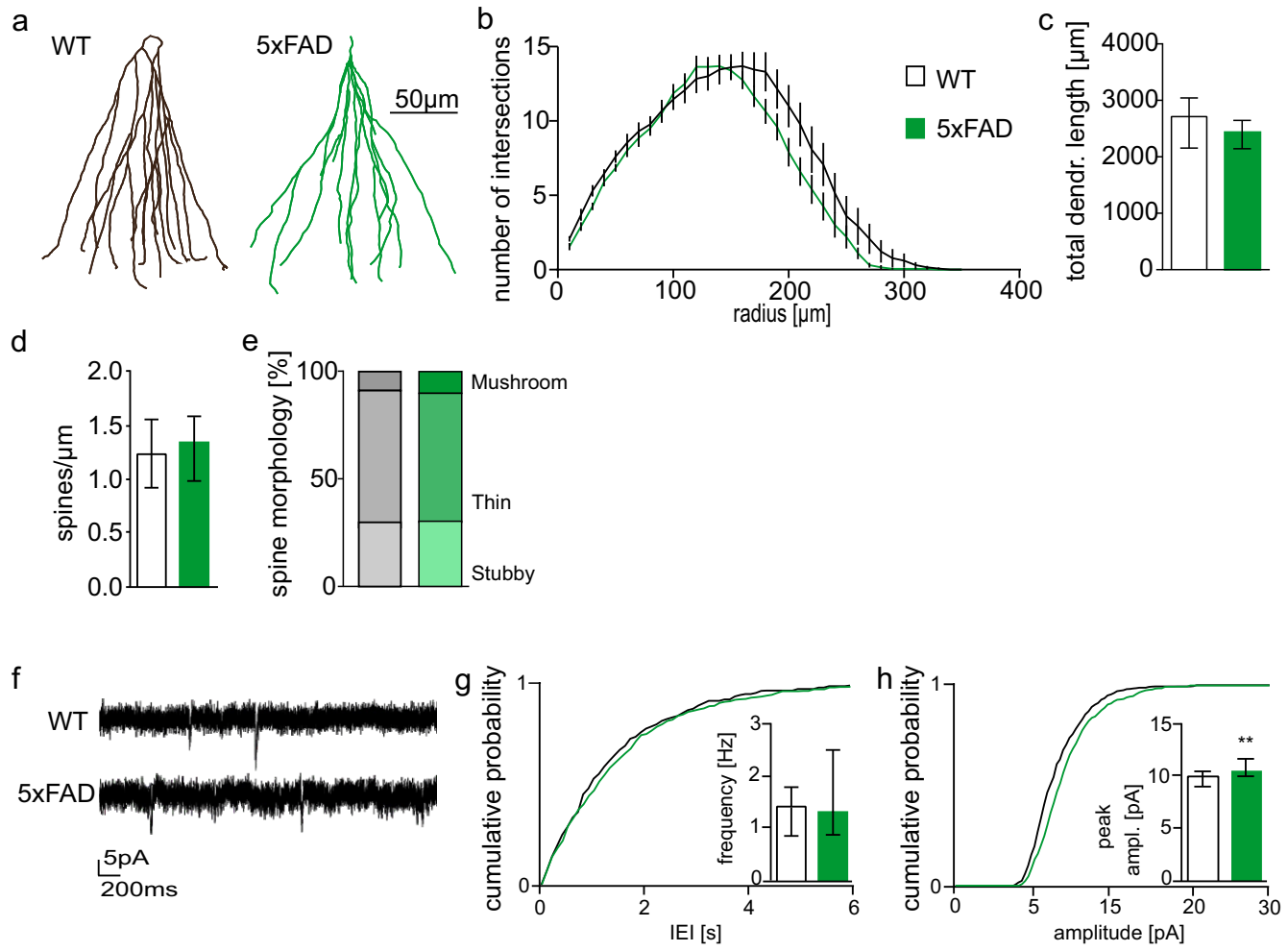

Supplement: Supplementary file 6 — S6. Functional and structural properties are not affected in six-month old 5xFAD mice. a Examples of traced DG granule cells of six-month old WT and 5xFAD mice. b The number of intersections per radius is not changed as revealed by a Sholl analysis of cells from 5xFAD and WT mice. Mean ± SEM. c Total dendritic length is also not changed. d + e Spine number and spine morphology is not affected in DG granule cells of 5xFAD compared to WT mice. f mEPSC example traces of WT and 5xFAD granule cells. g + h mEPSC frequency is not changed in 5xFAD compared to WT granule cells, but peak amplitude is increased. Bar graphs show median ± IQR. * = p < 0.05, ** = p < 0.01, *** = p < 0.001; dendr. = dendritic, morph. = morphology (PDF 100 kb) [file 40478_2018_611_MOESM6_ESM.pdf]
