# Supplementary material for: NMDA receptors mediate synaptic depression, but not spine loss in the dentate gyrus of adult amyloid Beta (Aβ) overexpressing mice
Source: Acta Neuropathol Commun. 2018 Oct 23;6:110. doi: 10.1186/s40478-018-0611-4 (PMC6198500; doi:10.1186/s40478-018-0611-4)
Supplement: Supplementary file 7 — Table S1. mEPSC recordings of CT100-overexpressing DG granule cells. Table S2. Morphological analysis of CT100-overexpressing DG granule cells. Table S3. mEPSC recordings of CT100(I716F)-overexpressing DG granule cells. Table S4. Morphology of CT100(I716F)-overexpressing DG granule cells. Table S5. Values of PPR of CT100(I716F)-overexpressing DG granule cells. Table S6. mEPSC recordings from the 5xFAD mouse model. Table S7. Morphological analysis of the 5xFAD mouse model. Table S8. Intrinsic and firing properties of CT100(I716F) overexpressing DG granule cells. Table S9. NMDAR-mediated currents in 5xFAD DG granule cells and virus-infected cells. Table S10. Values for spine morphology in CT100 and CT100(I716F) overexpression experiments. Table S11. Overview of values for spine morphology in 5xFAD mice. (DOCX 49 kb) [file 40478_2018_611_MOESM7_ESM.docx]

**Table S1: mEPSC recordings of CT100-overexpressing DG granule cells**

| **Adult mice** | | | | | |
| --- | --- | --- | --- | --- | --- |
| **3w pi** | **Control (n=22)** | | **CT100 (n=11)** | |  |
| **Frequency [Hz]** | 0.59 [0.37-0.77] | | 0.48 [0.44-0.71] | | MW test: p=0.9074 |
| **10w pi** | **Control (n=40)** | | **CT100 (n=19)** | |  |
| **Frequency [Hz]** | 0.71 [0.4 – 0.92] | | 0.54 [0.42 – 0.77] | | MW test p=0.21 |
| **Young mice** | | | | | |
| **9w pi** | **Control (n=56)** | | **CT100 (n=26)** | |  |
| **Frequency [Hz]** | 0.81 [0.51-1.02] | | 0.61 [0.41-0.81] | | MW test: p=0.047 |
| **GluN1^fl/fl^ 9w pi** | **Control (n=34)** | **CT100 (n=10)** | **GluN1^-/-^ (n=21)** | **GluN1^-/-^+CT100 (n=9)** |  |
| **Frequency [Hz]** | 0.69 [0.55 – 0.83] | 0.37 [0.32 – 0.55] | 0.99 [0.76 – 1.22] | 1.18 [1.08 – 1.47] | Kruskal-Wallis: p<0.0001; Dunn’s posttest: control vs CT100 p=0.04; control vs GluN1^-/-^ p=0.0045; GluN1^-/-^ vs GluN1^-/-^+CT100 p=0.7895 |
| **Amplitude [pA]** | 10.47 [9.27-11.53] | 10.47 [9.94-11.53] | 14.85 [12.96-16.09] | 12.4 [10.99-13.81] | Kruskal-Wallis: p<0.0001; Dunn’s posttest: control vs CT100 p>0.9999; control vs GluN1^-/-^ p<0.0001; GluN1^-/-^ vs GluN1^-/-^+CT100 p=0.336 |

**Table S2: Morphological analysis of CT100-overexpressing DG granule cells**

| **GluN1^fl/fl^ 9w pi** | | | | | |
| --- | --- | --- | --- | --- | --- |
| **Spine numbers** | **Control (n=23)** | **CT100 (n=6)** | **GluN1^-/-^ (n=23)** | **GluN1^-/-^+CT100 (n=10)** |  |
|  | 1.54 [1.25-1.84] | 2.22 [1.95-2.37] | 1.73 [1.54-1.9] | 1.51 [1.34-1.62] | Kruskal-Wallis: p=0.0015; Dunn’s posttest: control vs CT100 p=0.0026; control vs GluN1^-/-^ p=0.1332; GluN1^-/-^ vs GluN1^-/-^+CT100 p=0.1119 |

**Table S3: mEPSC recordings of CT100(I716F)-overexpressing DG granule cells**

| **GluN1^fl/fl^** | **Control (n=26)** | **CT100(I716F) (n=33)** | **GluN1^-/-^ (n=24)** | **GluN1^-/-^+CT100(I716F) (n=21)** |  |
| --- | --- | --- | --- | --- | --- |
| **Frequency [Hz]** | 0.66 [0.52-0.77] | 0.42 [0.3-0.64] | 0.89 [0.69-1.63] | 1.02 [0.68-1.23] | Kruskal-Wallis: p<0.0001; Dunn’s posttest: control vs CT100(I716F) p=0.029; control vs GluN1^-/-^ p=0.044; GluN1^-/-^ vs GluN1^-/-^+CT100(I716F) p>0.99 |
| **Percentual reduction** |  | 0.36 [0.02-0.55] |  | -0.15 [-0.38-0.23] | p=0.0137 |
| **Amplitude [pA]** | 10.81 [10.03-11.47] | 10.62 [10.02-11.13] | 11.2 [10.34-12.44] | 11.05 [9.78-12] | Kruskal-Wallis: p=0.3076; Dunn’s posttest: control vs CT100(I716F) p>0.99; control vs GluN1^-/-^ p=0.75; GluN1^-/-^ vs GluN1^-/-^+CT100(I716F) p>0.99 |
| **GluN2A^fl/fl^** | **Control (n=36)** | **CT100(I716F) (n=37)** | **GluN2A^-/-^ (n=24)** | **GluN2A^-/-^+CT100(I716F) (n=19)** |  |
| **Frequency [Hz]** | 0.61 [0.5-0.75] | 0.5 [0.31-0.68] | 0.76 [0.6-0.92] | 0.62 [0.46-0.85] | Kruskal-Wallis: p=0.0004; Dunn’s posttest: control vs CT100(I716F) p=0.0468; control vs GluN2A^-/-^ p=0.149; GluN2A^-/-^ vs GluN2A^-/-^+CT100(I716F) p=0.485 |
| **Percentual reduction** |  | 0.19 [-0.1-0.51] |  | 0.18 [-0.11-0.39] | p=0.27 |
| **Amplitude [pA]** | 9.67 [8.46-10.32] | 9.83 [9.3-10.82] | 10.47 [9.29-12.96] | 10.44 [9.84-11.95] | Kruskal-Wallis: p=0.013; Dunn’s posttest: control vs CT100(I716F) p=0.844; control vs GluN2A^-/-^ p=0.75; GluN2A^-/-^ vs GluN2A^-/-^+CT100(I716F) p>0.99 |
| **GluN2B^fl/fl^** | **Control (n=28)** | **CT100(I716F) (n=25)** | **GluN2B^-/-^ (n=27)** | **GluN2B^-/-^+CT100(I716F) (n=26)** |  |
| **Frequency [Hz]** | 0.71 [0.53-1.08] | 0.39 [0.28-0.75] | 1.01 [0.81-1.23] | 0.87 [0.72-1.03] | Kruskal-Wallis: p<0.0001; Dunn’s posttest: control vs CT100(I716F) p=0.013; control vs GluN2B^-/-^ p=0.018; GluN2B^-/-^ vs GluN2B^-/-^+CT100(I716F) p=0.497 |
| **Percentual reduction** |  | 0.45 [-0.06-0.6] |  | 0.14 [-0.02-0.29] | p=0.1 |
| **Amplitude [pA]** | 9.58 [8.61 – 10.26] | 9.65 [8.43 – 10.51] | 9.8 [9.2 – 10.61] | 11.59 [10.16 – 12.69] | Kruskal-Wallis: p<0.0001; Dunn’s posttest: control vs CT100(I716F) p>0.9999; control vs GluN2B^-/-^ p>.0.9999; GluN2B^-/-^ vs GluN2B^-/-^+CT100(I716F) p=0.0033 |

**Table S4: Morphology of CT100(I716F)-overexpressing DG granule cells**

| **GluN1^fl/fl^** | | | | | |
| --- | --- | --- | --- | --- | --- |
| **Spine numbers** | **Control (n=51)** | **CT100(I716F) (n=20)** | **GluN1^-/-^ (n=22)** | **GluN1^-/-^+CT100(I716F) (n=28)** |  |
|  | 1.7 [1.45 – 1.97] | 1.96 [1.69 – 2.27] | 1.46 [1.16 – 1.72] | 1.51 [1.23 – 1.98] | Kruskal-Wallis: p=0.0008; Dunn’s posttest: control vs CT100 p=0.1308 control vs GluN1^-/-^ p=0.0381; GluN1^-/-^ vs GluN1^-/-^+CT100 p>0.99 |
| **Total dendritic length [µm]** | **Control (n=15)** | **CT100(I716F) (n=27)** | **GluN1^-/-^ (n=17)** | **GluN1^-/-^+CT100(I716F) (n=22)** |  |
|  | 2106 [1843-2325] | 2155 [2018-2533] | 2090 [1782-2418] | 2019 [1495-2343] | Kruskal-Wallis: p=0.0195; Dunn’s posttest: control vs CT100 p>0.99 control vs GluN1^-/-^ p>0.99; GluN1^-/-^ vs GluN1^-/-^+CT100 p>0.99 |
| **GluN2A^fl/fl^** |  |  |  |  |  |
| **Spine numbers** | **Control (n=11)** | **CT100(I716F) (n=17)** | **GluN2A^-/-^(n=26)** | **GluN2A^-/-^+CT100(I716F) (n=21)** |  |
|  | 1.81 [1.56 – 1.09] | 2.97 [1.84 – 2.25] | 1.65 [1.42 – 1.95] | 1.67 [1.46 – 1.96] | Kruskal-Wallis: p=0.0015; Dunn’s posttest: control vs CT100 p=0.3366 control vs GluN2A^-/-^ p=0.5077; GluN2A^-/-^ vs GluN2A^-/-^+CT100 p>0.9999 |
| **Total dendritic length [µm]** | **Control (n=16)** | **CT100(I716F) (n=17)** | **GluN2A^-/-^(n=17)** | **GluN2A^-/-^+CT100(I716F) (n=18)** |  |
|  | 2162 [1657 – 2391] | 1889 [1577 – 2155] | 1862 [1528 – 2254] | 2046 [1885 – 2189] | Kruskal-Wallis: p=0.337; Dunn’s posttest: control vs CT100 p=0.5642 control vs GluN2A^-/-^ p=0.6218; GluN2A^-/-^ vs GluN2A^-/-^+CT100 p=0.6027 |
| **GluN2B^fl/fl^** |  |  |  |  |  |
| **Spine numbers** | **Control (n=31)** | **CT100(I716F) (n=45)** | **GluN2B^-/-^(n=29)** | **GluN2B^-/-^+CT100(I716F) (n=16)** |  |
|  | 1.91 [1.8-2.2] | 1.88 [1.65-2.13] | 1.63 [1.29-2.06] | 1.55 [1.18-1.87] | Kruskal-Wallis: p=0.0021; Dunn’s posttest: control vs CT100 p=0.7884 control vs GluN2B^-/-^ p=0.0151; GluN2B^-/-^ vs GluN2B^-/-^+CT100 p>0.99 |
| **Total dendritic length [µm]** | **Control (n=29)** | **CT100(I716F) (n=26)** | **GluN2B^-/-^(n=23)** | **GluN2B^-/-^+CT100(I716F) (n=16)** |  |
|  | 2248 [2013 – 2577] | 2223 [1882 – 2364] | 2336 [2064 – 2681] | 1882 [1668 – 2437] | Kruskal-Wallis: p=0.1092; Dunn’s posttest: control vs CT100 p=0.6198 control vs GluN2B^-/-^ p>0.9999; GluN2B^-/-^ vs GluN2B^-/-^+CT100 p=0.1049 |

**Table S5: Values of PPR of CT100(I716F)-overexpressing DG granule cells**

|  | **WT (n=20)** | **CT100(I716F) (n=20)** |  |
| --- | --- | --- | --- |
| **25ms ISI** | 0.84 [0.78-0.88] | 0.81 [0.74-0.87] | MW-test: p=0.2423 |
| **50ms ISI** | 1.1 [1.05-1.17] | 1.13 [1.07-1.26] | MW-test: p=0.201 |

**Table S6:mEPSC recordings from the 5xFAD mouse model**

| **6m DG** | | | |
| --- | --- | --- | --- |
|  | **WT (n=24)** | **5xFAD (n=23)** |  |
| **Frequency [Hz]** | 0.73 [0.44-0.91] | 0.66 [0.45-1.2] | MW-test: p=0.6612 |
| **Amplitude [pA]** | 10.08 [9.15 – 10.52] | 10.64 [10.15-11.7] | MW-test: p=0.0013 |
| **1a DG** | | | |
|  | **WT (n=27)** | **5xFAD (n=21)** |  |
| **Frequency [Hz]** | 0.80 [0.61-1.07] | 0.61 [0.44-0.89] | MW-test: p=0.026 |
| **Amplitude [pA]** | 10.44 [8.54 – 11.95] | 11.2 [8.94 – 11.86] | MW-test: p=0.47 |
|  | **GluN1^-/-^ (n=17)** | **5xFAD/ GluN1^-/-^ (n=17)** |  |
| **Frequency [Hz]** | 0.85 [0.68-1.26] | 1.02 [0.55-1.56] | MW-test: p=0.8119 |
| **Amplitude [pA]** | 11.2 [10.24 – 12.84] | 10.43 [9.66 – 12.31] | MW-test: p=0.394 |
|  | **GluN2A^-/-^ (n=23)** | **5xFAD/ GluN2A^-/-^ (n=17)** |  |
| **Frequency [Hz]** | 0.97 [0.61-1.13] | 1.01 [0.64-1.98] | MW-test: p=0.2802 |
| **Amplitude [pA]** | 9.0 [8.38 – 9.93] | 9.45 [8.97 – 10.72] | MW-test: p=0.1626 |
|  | **GluN2B^-/-^ (n=21)** | **5xFAD/ GluN2B^-/-^ (n=16)** |  |
| **Frequency [Hz]** | 1.26 [0.89-1.61] | 1.31 [0.6-1.63] | MW-test: p>0.999 |
| **Amplitude [pA]** | 11.63 [10.72 – 12.14] | 10.06 [9.88 – 12.92] | MW-test: p=0.3232 |

**Table S7:Morphological analysis of the 5xFAD mouse model**

| **6m DG** | | | |
| --- | --- | --- | --- |
| **Spine numbers** | **WT (n=17)** | **5xFAD (n=20)** |  |
|  | 1.25 [0.93 – 1.56] | 1.36 [1 – 1.6] | MW-test: p=0.8923 |
| **Total dendritic length** | **WT (n=13)** | **5xFAD (n=20)** |  |
|  | 2707 [2131 – 3003] | 2425 [2134 – 2630] | MW-test: p=0.1275 |
| **1a DG** | | | |
| **Spine numbers** | **WT (n=27)** | **5xFAD (n=28)** |  |
|  | 1.54 [1.35-1.99] | 1.07 [0.73-1.46] | MW-test: p<0.0001 |
|  | **GluN1^-/-^ (n=6)** | **5xFAD/GluN1^-/-^ (n=12)** |  |
|  | 1.62 [1.36-1.76] | 1.13 [0.96-2.02] | MW-test: p=0.325 |
|  | **GluN2A^-/-^ (n=12)** | **5xFAD/GluN2A^-/-^ (n=10)** |  |
|  | 1.7 [1.36-1.98] | 0.87 [0.64-1.04] | MW-test: p=0.0001 |
|  | **GluN2B^-/-^ (n=28)** | **5xFAD/GluN2B^-/-^ (n=13)** |  |
|  | 1.73 [1.29-1.96] | 1.33 [1.02-1.6] | MW-test: p=0.0165 |
| **Total dendritic length [µm]** | **WT (n=22)** | **5xFAD (n=19)** |  |
|  | 2222 [1704 – 2660] | 1882 [1708 – 2480] | MW-test: p=0.4763 |

**Table S8: Intrinsic and firing properties of CT100(I716F) overexpressing DG granule cells**

|  | **3w pi CT100(I716F)** | | |
| --- | --- | --- | --- |
|  | **Control** | **CT100(I716F)** |  |
|  | n=31 | n=20 |  |
| **Passive properties** |  |  |  |
| **Input resistance [mΩ]** | 182 [140-211.5] | 170 [129.5-184] | MW test: p=0.2418 |
| **Active properties** |  |  |  |
| **AP threshold [mV]** | -37.27 [-39.18 - -33.78] | -35.84 [-39.04 - -30.2] | MW test: p=0.5246 |
| **AP width [ms]** | 1.26 [1.2-1.32] | 1.24 [1.15-1.28] | MW test: p=0.3286 |
| **AP amplitude [mV]** | 94.03 [90.88-97.7] | 91.25 [87.12-95.74] | MW test: p=0.1308 |
| **AHP [mV]** | -13.83 [-16-58- -10] | -13.76 [-15.77- - 11.23] | MW test: p=0.7964 |
| **Firing properties** |  |  |  |
| **Firing frequency [Hz]** | 22 [16-26] | 20.5 [17.25-23.75] | MW test: p=0.7484 |
| **Early adaptation [%]** | 451.7 [356-563.4] | 391.4 [347.1-543.1] | MW test: p=0.6064 |
| **Late adaptation [%]** | 41.98 [24.16 – 61.51] | 42.37 [20.43-102.4] | MW test: p=0.8231 |

**Table S9:NMDAR-mediated currents in 5xFAD DG granule cells and virus-infected cells**

|  | **Control (n=16)** | **GluN1^-/-^ (n=15)** |  |
| --- | --- | --- | --- |
| **NMDAR/AMPAR ratio** | 1 ± 0.65 | 0.13 ± 0.04 | MW test: p<0.0001 |
|  | **WT (n=22)** | **5xFAD (n=29)** |  |
| **NMDAR/AMPAR ratio** | 1.18 [0.79-1.77] | 0.72 [0.43-1.2] | MW test: p=0.0029 |
|  | **WT (n=18)** | **5xFAD (n=25)** |  |
| **Decay tau [ms]** | 62.91 [57.75-67.48] | 66.51 [59.2-72.86] | MW test: p=0.0969 |
|  | **WT (n=23)** | **5xFAD (n=22)** |  |
| **Extrasynaptic amplitude [pA]** | 125.3 [85.8-178.6] | 77.57 [43.12-101.2] | MW test: p=0.0003 |
|  | **WT (n=23)** | **5xFAD (n=22)** |  |
| **Deactivation [ms]** | 74.76 [63.62-88.33] | 79.43 [71.22-104.3] | MW test: p=0.1712 |

**Table S10: Values for spine morphology in CT100 and CT100(I716F) overexpression experiments**

|  | **Spine morphology distribution [%]** | | |
| --- | --- | --- | --- |
|  | **Stubby** | **Thin** | **Mushroom** |
| 9w pi CT100 in P7 floxed GluN1 | | | |
| Control (23) | 0.29 [0.26-0.31] | 0.62 [0.6-0.67] | 0.09 [0.47-0.11] |
| CT100 (6) | 0.36 [0.27-0.4] | 0.59 [0.54-0.64] | 0.06 [0.04-0.09] |
| GluN1^-/-^ (10) | 0.29 [0.23-0.35] | 0.6 [0.57-0.7] | 0.07 [0.05-0.1] |
| GluN1^-/-^+CT100 (23) | 0.27 [0.23-0.32] | 0.64 [0.62-0.67] | 0.07 [0.05-0.11] |
| Kruskal Wallis test (Dunn’s posttest) | P=0.1972 (Control vs CT100: p=0.4813 Control vs GluN1-/: p>0.9999; GluN1-/- vs GluN1-/-+CT100: p>0.9999) | P=0.1433 (Control vs CT100: p=0.6288; Control vs GluN1^-/^: p>0.9999; GluN1^-/-^ vs GluN1^-/-^+CT100: p=0.5863) | P=0.8439 (Control vs CT100: p>0.9999; Control vs GluN1^-/^: p>0.9999; GluN1^-/-^ vs GluN1^-/-^+CT100: p>0.9999) |
| DG granule cells GluN1^-/-^ line | | | |
| Control (49) | 0.32 [0.27-0.36] | 0.61 [0.54-0.64] | 0.08 [0.05-0.10] |
| CT100(I716F) (19) | 0.29 [0.27-0.33] | 0.63 [0.59-0.66] | 0.08 [0.06-0.08] |
| GluN1^-/-^ (22) | 0.32 [0.26-0.39] | 0.56 [0.49-0.64] | 0.1 [0.06-0.14] |
| GluN1^-/-^+CT100(I716F) (28) | 0.32 [0.27-0.37] | 0.61 [0.55-0.67] | 0.07 [0.05-0.85] |
| Kruskal Wallis test (Dunn’s posttest) | p=0.529 (Control vs CT100(I716F): p=0.53; Control vs GluN1^-/-^: p>0.9999; GluN1^-/-^ vs GluN1^-/-^+CT100(I716F): p>0.9999) | p=0.198 (Control vs CT100(I716F): p=0.5339; Control vs GluN1^-/-^: p=0.8345; GluN1^-/-^ vs GluN1^-/-^+CT100(I716F): p=0.3877) | p=0.1098 (Control vs CT100(I716F): p>0.9999; Control vs GluN1^-/-^: p=0.2132; GluN1^-/-^ vs GluN1^-/-^+CT100(I716F): p=0.0511) |
| DG granule cells GluN2A^fl/fl^ line | | | |
| Control (11) | 0.35 [0.32-0.37] | 0.57 [0.53-0.62] | 0.08 [0.03-0.11] |
| CT100(I716F) (17) | 0.36 [0.29-0.38] | 0.57 [0.52-0.62] | 0.07 [0.06-0.11] |
| GluN2A^-/-^ (26) | 0.38 [0.34-0.42] | 0.54 [0.49-0.58] | 0.1 [0.06-0.18] |
| GluN2A^-/-^+CT100(I716F) (21) | 0.34 [0.3-0.38] | 0.55 [0.52-0.61] | 0.1 [0.08-0.13] |
| Kruskal Wallis test (Dunn’s posttest) | p=0.1208 (Control vs CT100(I716F): p>0.999; Control vs GluN1^-/-^: p=0.9455; GluN1^-/-^ vs GluN1^-/-^+CT100(I716F): p=0.0586) | p=0.2321 (Control vs CT100(I716F): p>0.9999; Control vs GluN1^-/-^: p=0.2893; GluN1^-/-^ vs GluN1^-/-^+CT100(I716F): p=0.7813) | p>0.1487 (Control vs CT100(I716F): p>0.9999; Control vs GluN1^-/-^: p>0.9999; GluN1^-/-^ vs GluN1^-/-^+CT100(I716F): p=0.4372) |
| DG granule cells GluN2B^fl/fl^ line | | | |
| Control (31) | 0.36 [0.33-0.42] | 0.55 [0.49-0.58] | 0.08 [0.06-0.11] |
| CT100(I716F) (45) | 0.32 [0.28-0.4] | 0.59 [0.53-0.6442] | 0.07 [0.05-0.1] |
| GluN2B^-/-^ (29) | 0.33 [0.28-0.39] | 0.56 [0.49-0.6] | 0.11 [0.09-0.14] |
| GluN2B^-/-^+CT100(I716F) (16) | 0.37 [0.33-0.44] | 0.57 [0.5-0.6] | 0.07 [0.04-0.09] |
| Kruskal Wallis test (Dunn’s posttest) | P=0.0105 (Control vs CT100(I716F): p=0.0277; Control vs GluN2B^-/-^: p=0.043; GluN2B^-/-^ vs GluN2B^-/-^+CT100(I716F): p=0.1063) | P=0.0319 (Control vs CT100(I716F): p=0.0112; Control vs GluN2B^-/-^: p=0.6837; GluN2B^-/-^ vs GluN2B^-/-^+CT100(I716F): p>0.9999) | P=0.0002 (Control vs CT100(I716F): p>0.9999; Control vs GluN2B^-/-^: p=0.0066; GluN2B^-/-^ vs GluN2B^-/-^+CT100(I716F): p=0.0048) |

**Table S11: Overview of values for spine morphology in 5xFAD mice**

|  | **Spine Morphology distribution [%]** | | |
| --- | --- | --- | --- |
|  | **Stubby** | **Thin** | **Mushroom** |
| **6m 5xFAD DG** | | | |
| WT (17) | 0.31 [0.25-0.34] | 0.6 [0.56-0.64] | 0.1 [0.06-0.1] |
| 5xFAD (18) | 0.32 [0.27-0.35] | 0.58 [0.56-0.61] | 0.1 [0.09-0.11] |
| Mann-Whitney test | p=0.4 | p=0.142 | p=0.85 |
| **1a 5xFAD DG** | | | |
| WT (29) | 0.26 [0.22-0.35] | 0.64 [0.6-0.7] | 0.08 [0.04-0.11] |
| 5xFAD (28) | 0.32 [0.23-0.38] | 0.59 [0.23-0.38] | 0.09 [0.06-0.16] |
| Mann-Whitney test | p=0.32 | p=0.13 | p=0.14 |
| GluN1^-/-^ (7) | 0.32 [0.27-0.39] | 0.56 [0.45-0.62] | 0.12 [0.12-0.16] |
| 5xFAD/GluN21^-/-^ (12) | 0.29 [0.25-0.31] | 0.57 [0.47-0.63] | 0.14 [0.12-0.21] |
| Mann-Whitney test | p=0.16 | p=0.526 | p=0.29 |
| GluN2A^-/-^ (12) | 0.31 [0.24-0.36] | 0.58 [0.47-0.64] | 0.13 [0.09-0.17] |
| 5xFAD/GluN2A^-/-^ (10) | 0.39 [0.32-0.5] | 0.39 [0.29-0.46] | 0.19 [0.14-0.26] |
| Mann-Whitney test | p=0.02 | p=0.002 | p=0.025 |
| GluN2B^-/-^ (15) | 0.35 [0.27-0.4] | 0.53 [0.45-0.62] | 0.1 [0.08-0.14] |
| 5xFAD/GluN2B^-/-^ (11) | 0.42 [0.34-0.52] | 0.47 [0.37-0.54] | 0.13 [0.1-0.16] |
| Mann-Whitney test | p=0.0362 | p=0.0687 | p=0.3565 |
